# A new approach of a path-tracking system of a differential drive robot for an arbitrary set of waypoints using sliding mode control

**DOI:** 10.1371/journal.pone.0357979

**Published:** 2026-10-02

**Authors:** Amin Danial Asham

**Affiliations:** Egyptian Academy for Engineering and Advanced Technology, Cairo, Egypt; Beijing Institute of Technology, CHINA

## Abstract

Autonomous differential-drive robots are typically controlled to follow a desired path. This path may be defined by a continuous curve or set of waypoints. Few studies have examined cases in which a path is defined by an arbitrary set of waypoints. This paper introduces a new approach to a path-tracking system for an arbitrary set of waypoints using a *sliding mode control* (SMC) system. This approach is based on a nonlinear kinematic model of the tracking error derived in this research for a differential-drive robot. Based on the derived error model, a sliding mode controller is introduced to drive the tracking error to zero. The stability of the system was also investigated. In addition, the effects of robot dynamics were incorporated into the proposed controller without prior knowledge of the exact parameters of the robot. Simulations were conducted to compare the proposed control system with a widely used approach to demonstrate the effectiveness of the proposed controller in tight maneuvers, particularly at directional discontinuity and/or sharp turns. The proposed technique was verified through real-time experimental tests using a differential drive robot.

## 1. Introduction

Autonomous robots have a wide range of applications in various fields. These robots are typically used to track desired paths. If paths are drawn as lines and detected by sensors, this is called the line-following approach, as in [1]. For paths defined by smooth curves or waypoints, this approach is called the path-tracking approach. The path is planned based on the environment in which the robot is moving. Path-tracking algorithms are more complex than line-follower systems. A line-follower system can be viewed as a special case of a path-tracking system. In [2], a kinematic model was developed for the path-tracking error of a differential-drive robot. Based on this model, the target velocities are calculated to reduce the tracking error to zero. These target velocities are applied to the robot to track the desired path. However, the effect of robot dynamics was ignored by assuming that the robot was capable of perfectly tracking the target velocities. In addition, the desired path in [2] is a piecewise continuous function of time and not a set of waypoints. Moreover, the proposed controller has a cross-track error (perpendicular lateral distance from the desired path) in the case of a desired path with directional discontinuity. The kinematic controller introduced in [2] is widely used in the literature, as in [3–6]. In [3], a backstepping control approach was introduced to address the dynamics of real robots. In this approach, a dynamic controller was added to achieve the target velocities from the kinematic controller introduced in [2]. The control scheme introduced in [3] has been used widely by several researchers. That is, the target velocities were generated as in [2] and another controller was used to drive the real velocities of the robot to follow the target velocities. Different controllers have been introduced to achieve accurate tracking of target velocities, such as those in [4–6]. In [4], a modified PID controller was used to track the target velocities of a tracked vehicle. However, the proposed controller was not experimentally verified in a real vehicle. In [5], SMC was used to control the robot velocities to follow the target velocities. In addition, fuzzy logic was used to change a certain gain to reduce the chattering effect of the sliding mode control. In [6], a control scheme similar to that in [5] was employed. The control scheme consists of a kinematic controller and an integral SMC as a dynamic controller to deal with robot dynamics and uncertainties. The controller parameters were optimized using *sequential quadratic programming* (SQP). It was shown in [6] that the performance of the SMC is better than that of the modified PID controller [4]. All former approaches are derivatives of the control scheme introduced in [3]. This type of path tracking is primarily used for continuous, smooth paths. Various approaches have been proposed in the literature [7–12]. All these approaches are for continuous paths. Some approaches use fuzzy-based controllers [8,9]. Nonlinear model predictive control with neural networks was introduced in [10]. Even for discrete reference points, curve-fitting techniques have been used to obtain continuous smooth paths [7,11]. A feedback linearization method was used in [12], in which the derivation of the control laws assumed a differentiable path. A few researchers have introduced waypoint path-tracking algorithms [13–15]. In [13], the path was composed of a smooth polynomial connecting the waypoints. However, in [14], the proposed approach was a point-to-point control technique. The robot is controlled to reach each waypoint. However, no specific path existed between any two successive waypoints. In other words, the controller has no cross-track error measures to eliminate. This case was solved in [15], where the desired path was defined by a set of waypoints connected to each other with straight segments. A modified version of the kinematic controller introduced in [2] was developed in [15] by adding a cross-track error measure to be eliminated using the modified controller. This technique outperforms that in [2] for turns with directional discontinuities. However, another controller must be used to achieve the desired linear and angular velocities calculated using this modified kinematic controller. Moreover, the angular velocity control signal produced by this controller exhibits fluctuations that may limit its applicability in real-world applications. In addition, this controller does not perform tight maneuvers during sharp or directional discontinuous turns.

In this paper, a new approach for the path tracking of an arbitrary set of waypoints is introduced. This approach is based on the nonlinear kinematic model of the differential-drive robot derived in Section 2. An SMC scheme is introduced in Section 3, in which a sliding surface is selected to ensure that the tracking error approaches zero. The effect of robot dynamics is incorporated into the introduced controller. In Section 4, the proposed controller is tested through simulations and verified using a differential-drive robot. Finally, the conclusions of the results and future work are discussed in Section 5.

The main contribution of this research is the introduction of a new control approach for an arbitrary set of waypoints. This approach is based on a single sliding mode control method, taking into account both the kinematics and dynamics of the system. The proposed controller performs tight maneuvers at directional discontinuities and/or sharp turns (where a turning angle is considered sharp if it is ≥ π/2 radians) while accurately following the straight segments connecting the waypoints.

## 2. Kinematic mathematical model

In this section, the kinematic model of a differential-drive mobile robot is used as a foundation for the proposed path-tracking controller. While the basic pose kinematics rely on standard coordinates transformations the primary focus here is the derivation of a specialized error state model. This formulation, based on cross-track and angular errors, is explicitly tailored for tracking an arbitrary set of waypoints and allows the system to be treated as a single-input model for the subsequent sliding mode control design.

### 2.1. Standard differential-drive kinematics

The main objective is to drive a differential-drive robot to follow a path defined by an arbitrary set of waypoints, as shown in Fig 1. The robot must tightly follow straight segments that connect the waypoints. Fig 1 shows there are two frames: a fixed frame F−frame attached to the ground and a robot frame R−frame attached to the robot. The origin of the R−frame is located at point p, which is in the middle of the distance w between the powered wheels. The R−frame is rotated with respect to the F−frame with pose angle θ(t). The angular velocity of the robot is $\omega (t)=\dot{\theta }(t)$. The linear velocity ϑ(t) of the robot is in the x−axis direction of the R−frame.

**Fig 1:**
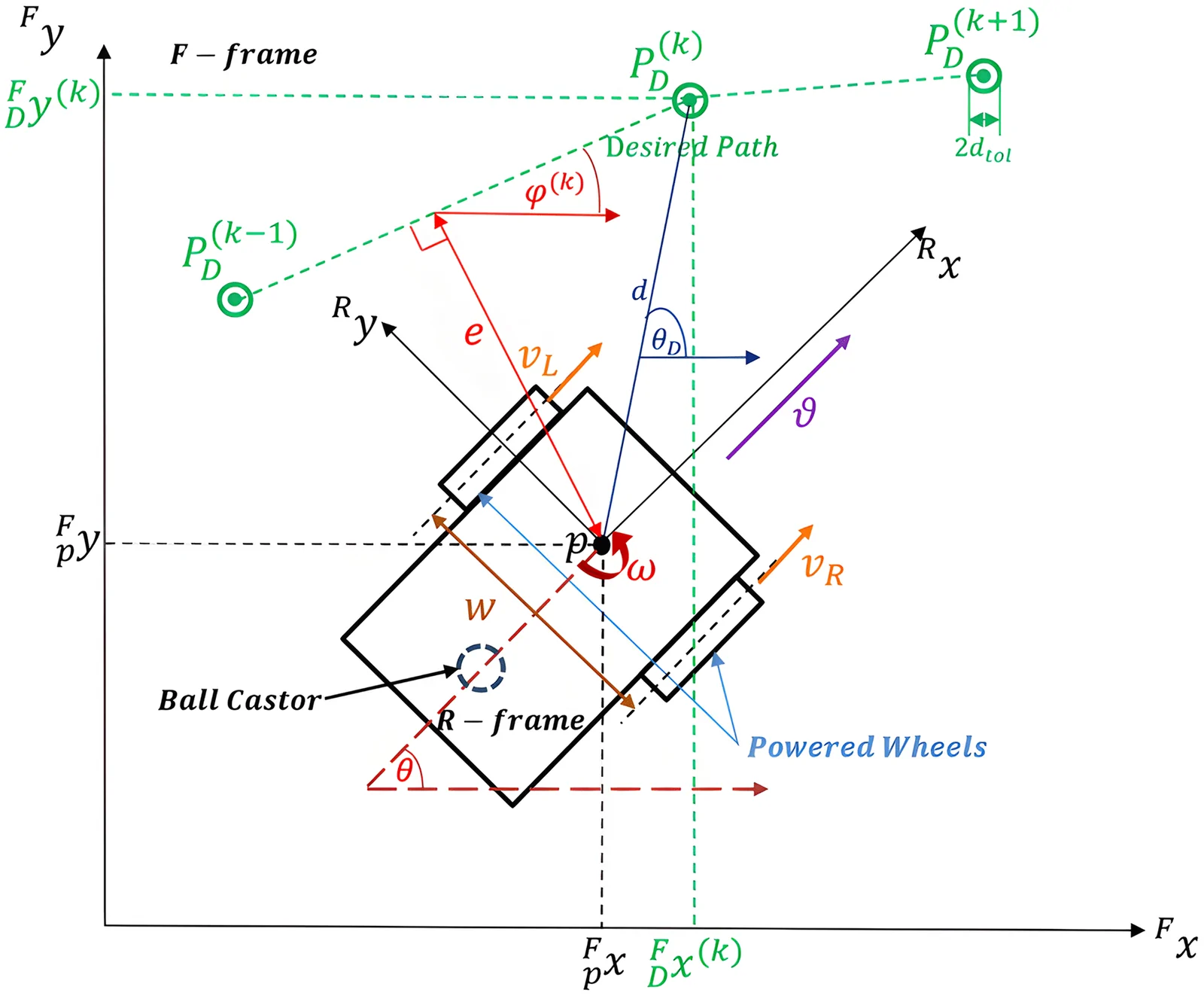
Differential drive robot. All the quantities $\backslash {(}_{p}^{F}x{,}_{p}^{F}y,\theta ,e,d,\;\omega ,\;\vartheta ,v_{R},and\;v_{L}$ are functions of time t, which is omitted for simplicity.

The coordinates of the point p in the fixed frame are XpFt. The desired path is defined as a sequence of desired waypoints in the fixed frame. These waypoints are connected by straight segments. The coordinates of the kth waypoint $P_{D}^{\left(k\right)}$in the F−frame are ${(}_{D}^{F}x^{\left(k\right)}{,}_{D}^{F}y^{\left(k\right)})$. As shown in Fig 1, the cross-track error e(t) is the perpendicular distance between the point p and the segment connecting the current waypoint $P_{D}^{\left(k\right)}$and the previous waypoint $P_{D}^{\left(k-\text{1}\right)}$. The angle between the segment connecting waypoints $P_{D}^{\left(k\right)}$ and $P_{D}^{\left(k-\text{1}\right)}$ and the x−axis of the fixed frame is φ. The distance between point p and the kth waypoint $P_{D}^{\left(k\right)}$ is xkDF-xpFt2+ykDF-ypFt2. The angle between d(t) and the x−axis in the F−frame is $\theta_{D}(t)$.

The linear velocity ϑ(t) and the angular velocity ω(t) are calculated as follows [16]:


$$\begin{array}{c} \vartheta (t)=\frac{v_{R}(t)+v_{L}(t)}{\text{2}} \end{array}$$

$$\begin{array}{c} \omega (t)=\frac{v_{R}(t)-v_{L}(t)}{𝑤} \end{array}$$ (1)

where $v_{R}(t)$ and $v_{L}(t)$ are the right and left linear velocities in m/s for the right and left wheels, respectively. The robot moves in the positive direction of the x−axis in the R−frame. That is, ϑ(t)≥0, in other words, both $v_{R}(t)$ and $v_{L}(t)$ never simultaneously become negative. If the maximum achievable speed by the powered wheels is $\vartheta_{m},$ then:


$$\begin{array}{c} \left|v_{R}(t)\right|\leq \vartheta_{m}\text{ and }\left|v_{L}(t)\right|\leq \vartheta_{m} \end{array}$$ (2)

Consequently, both the linear and angular velocities of the robot can be expressed as:


$$\begin{array}{c} \omega (t)=\omega_{m}\omega_{n}(t) \end{array}$$

$$\begin{array}{c} \vartheta (t)=\vartheta_{m}\vartheta_{n}(t)=\vartheta_{m}\left(\text{1}-\left|\omega_{n}(t)\right|\right) \end{array}$$ (3)

where $\omega_{m}$ is the maximum achievable angular velocity and $\omega_{n}(t)$ is the normalized angular velocity; hence, $\left|\omega_{n}(t)\right|\leq \text{1}$. $\vartheta_{n}(t)$ is the normalized linear velocity where $\vartheta_{n}(t)=\left(\text{1}-\left|\omega_{n}(t)\right|\right)$. From Eq. (1) and Eq. (3), we get:


$$\begin{array}{c} \omega_{\text{m}}\leq \frac{\text{2}\vartheta_{\text{m}}}{\text{w}} \end{array}$$ (4)

In the following subsection, a mathematical formulation of the cross-track and angular errors is introduced.

### 2.2. Proposed kinematic error model

The cross-track error e(t) in Eq. (5) is obtained from the dot product of two vectors, ${\left(-\text{sin}(\varphi ),\text{cos}(\varphi )\right)}^{T}$ and xkDF-xpFt,ykDF-ypFtT, where ${\left(-\text{sin}(\varphi ),\text{cos}(\varphi )\right)}^{T}$ is the unit vector perpendicular to the segment connecting the current waypoint $P_{D}^{\left(k\right)}$and the previous waypoint $P_{D}^{\left(k-\text{1}\right)}$.


et=-sinφ xkDF-xpFt + cosφ ,ykDF-ypFt (5)

Whereas the angular error $e_{\theta }(t)$ is defined as in Eq. (6), which is a modified version of the angular error in [15].


$$\begin{array}{c} e_{\theta }(t)=\left\{ \begin{array}{c} \theta_{D}(t)-\theta (t)-\text{2}\pi .\text{sign}(\theta_{D}(t)-\theta (t)),\;\left|\theta_{D}(t)-\theta (t)\right|>\pi \\ \theta_{D}(t)-\theta (t),\text{ otherwise} \end{array}\right. \end{array}$$ (6)

From Eq. (5), we get $\dot{e}(t)$ as follows:


e˙t=-sinφ x˙pFt - cosφ y˙pFt (7)

Since $\backslash {(}_{p}^{F}\dot{x}(t)=\vartheta (t)\text{cos}\left(\theta (t)\right)$ and $\backslash {(}_{p}^{F}\dot{y}(t)=\vartheta (t)sin\left(\theta (t)\right)$, then from Eq. (7), we get $\dot{e}(t)$ as follows:


$$\begin{array}{c} \dot{e}(t)=\vartheta (t)sin\left(\varphi -\theta (t)\right)=\vartheta (t)sin\left(\varphi -\theta_{D}(t)+e_{\theta }(t)\right) \end{array}$$ (8)

From Fig 1, $\theta_{D}(t)={\text{tan}}^{-\text{1}}\left(\frac{DFy^{\left(k\right)}{-}_{p}^{F}y(t)}{DFx^{\left(k\right)}{-}_{p}^{F}x(t)}\right)$, then from Eq. (6), we get:


$$\begin{array}{c} {\dot{e}}_{\theta }(t)\;={\dot{\theta }}_{D}(t)-\dot{\theta }(t)=\;\frac{\vartheta (t)}{d(t)}sin(\theta_{D}(t)-\theta (t))-\omega (t) \end{array}$$

$$\begin{array}{c} =\frac{\vartheta (t)}{d(t)}sin(e_{\theta }(t))-\omega (t) \end{array}$$ (9)

Then from Eq. (8) and Eq. (9), the proposed kinematic error model, incorporating cross-track and angular errors, can be expressed as follows:


$$\begin{array}{c} \dot{e}(t)=\vartheta (t)sin\left(\varphi -\theta_{D}(t)+e_{\theta }(t)\right) \end{array}$$

$$\begin{array}{c} {\dot{e}}_{\theta }(t)=\frac{\vartheta (t)}{d(t)}sin\left(e_{\theta }(t)\right)-\omega (t) \end{array}$$ (10)

From Eq. (10) and Eq. (3), the kinematic model becomes a single-input system with input control signal ω(t). Based on the kinematic model shown in Eq. (10), a control law is developed in the following section to obtain e(t)→0 and $e_{\theta }(t)\to \text{0}$ as t→∞.

## 3. Proposed path-tracking control system

In this section, a kinematic controller based on the SMC technique is proposed. This controller pushes the system to move on a certain sliding surface in the error state space $e-e_{\theta }$. This surface is chosen such that the tracking errors vanish with time. Hereafter, time variable t is omitted without ambiguity for simplicity. For example, e(t) is written as e, and so on.

### 3.1. Control law

The proposed kinematic controller is a sliding mode controller. This controller has a sliding surface σ in the state space $e-e_{\theta }$ defined as follows:


$$\begin{array}{c} \sigma =\frac{e_{n}+{e_{\theta }}_{n}}{\text{2}} \end{array}$$ (11)

where ${\text{e}}_{n}=e/e_{m}$ and ${e_{\theta }}_{n}=e_{\theta }/{e_{\theta }}_{m}$ are the normalized cross-track and angular errors, respectively. $e_{m}$and ${e_{\theta }}_{m}$ are the normalizing constants for the error e and angular error $e_{\theta }$, respectively. $e_{m}\;$and ${e_{\theta }}_{m}$can be interpreted as the maximum allowed errors for both the cross-track and the angular errors, respectively, at which the controller takes the maximum action. ${\text{1}/e}_{m}$ and 1/ ${e_{\theta }}_{m}$ represent the relative weights of both the cross-track and the angular errors, respectively. Consequently, these relative weights determine which error (cross-track or angular) has a greater impact on σ and hence on the control output. The error with higher impact will be prioritized for minimization. The proposed controller’s strategy is to push the system states (e and $e_{\theta })\;$towards the surface σ=0. On the surface σ=0, both $e\text{ and }e_{\theta }\;$tend to zero as t→∞ as proven by Theorem 1. Theorem 2 introduces a control law that pushes the states to surface σ=0 in finite time whenever σ≠0, ensuring that the states remain on surface σ=0. Theorem 3 is similar to Theorem 2 after considering robot dynamics.

**Theorem 1:** In case of σ=0 both $e,\;e_{\theta }\to \text{0}\text{ as }t\to \infty$.

**Proof:**

In the case of σ=0 and from Eq. (11), ${e_{\theta }}_{n}$ can be expressed as follows:


$$\begin{array}{c} {e_{\theta }}_{n}=-e_{n} \end{array}$$ (12)

From Fig 1, $\varphi -\theta_{D}(t)=-{\text{sin}}^{-\text{1}}(e/d)$, and substituting Eq. (12) into Eq. (10), the rate of change of the error is given by Eq. (13).


$$\begin{array}{c} \dot{e}=-\vartheta sin\left({sin}^{-1}\left(\frac{e}{d}\right)+\frac{{e_{\theta }}_{m}}{e_{m}}\;e\right) \end{array}$$ (13)

Let $V_{e}$ be a Lyapunov function candidate, defined as $V_{e}=(\text{1}/\text{2})e^{\text{2}}$. Then ${\dot{V}}_{e}=\dot{e}e$. Multiplying both sides of Eq. (13) with e, we find that $\dot{e}e<\text{0}$ and hence, ${\dot{V}}_{e}<\text{0}\;$for all e≠0. Consequently, e→0 and hence, from Eq. (12) $e_{\theta }\to \text{0}$ as t→∞. Therefore, the proof is complete.

As shown in Theorem 1, for σ=0 the system is asymptotically stable because the errors vanish as time t passes, as shown in Fig 2.

**Fig 2:**
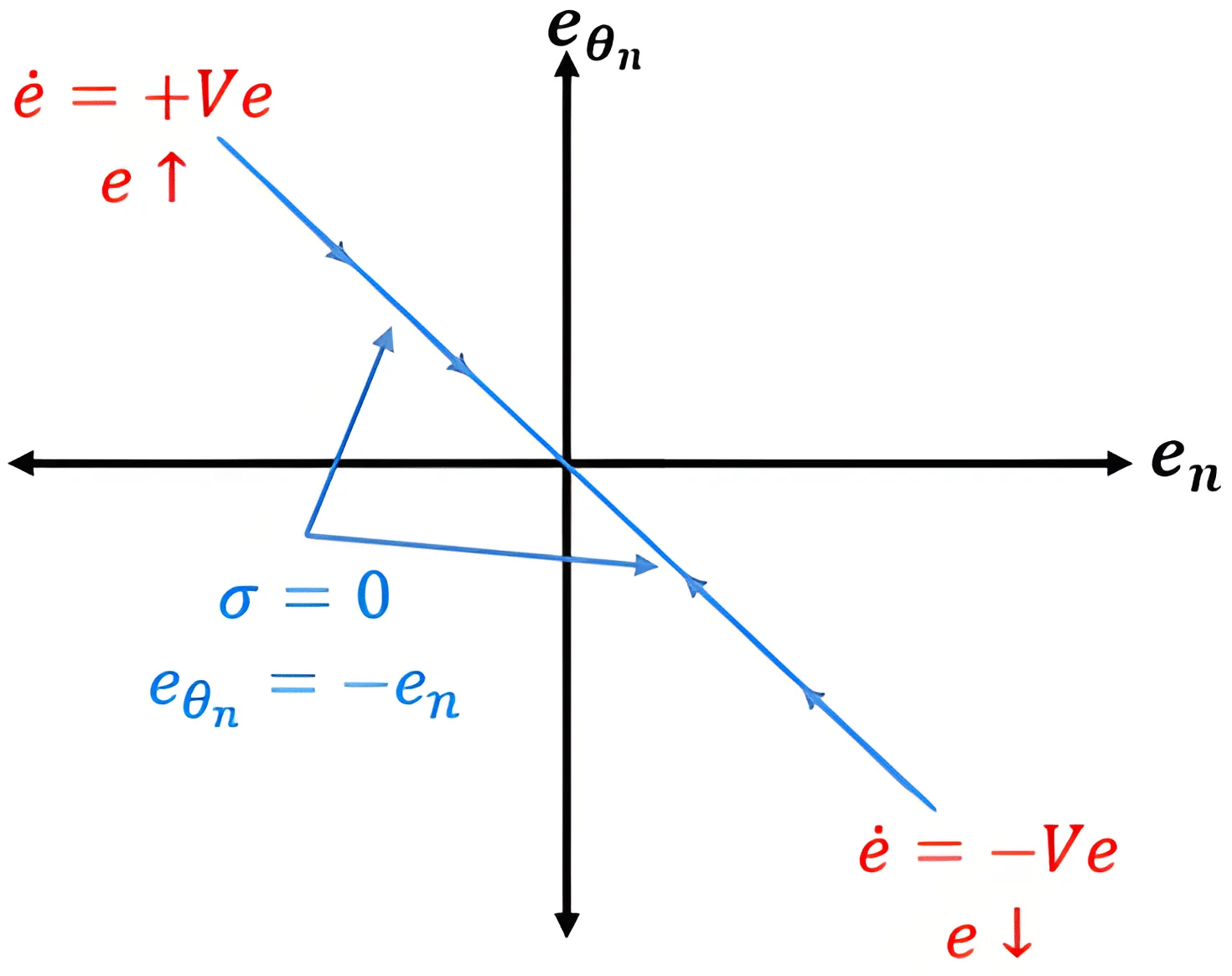
State Plane $e_{n}-e_{\theta_{n}}$. In the second and fourth quarters the error e→0. On σ=0, ${e_{\theta }}_{n}=-e_{n}$ and hence, ${e_{\theta }}_{n}\to 0\;as\;e_{n}\to 0$.

At this point, a control signal is required to push the system to σ=0. In Theorem 2, a control law is proposed that pushes the system towards σ=0.

**Theorem 2:** The Control law in Eq. (14) guarantees the reachability of the sliding surface in finite time and remains on it.


$$\begin{array}{c} \omega_{n}=\left\{ \begin{array}{cc} \text{0}, & \left|N|>D\;AND\;sign(N)=-sign(\sigma \right)\\ sign(\sigma ), & D^{\prime }=\text{0}\\ \frac{N^{\prime }}{D^{\prime }}, & otherwise \end{array}\right. \end{array}$$ (14)

where


$$\begin{array}{c} N=\frac{\vartheta_{m}}{\text{2}e_{m}}\left(sin(\varphi -\theta )+\frac{e_{m}}{e_{\theta_{m}}}\frac{sin\left(e_{\theta }\right)}{d}\right),N^{\prime }=cN+Dsign(\sigma )\sqrt{\left|\sigma \right|}, \end{array}$$

$$\begin{array}{c} D=\frac{\omega_{m}}{\text{2}e_{\theta_{m}}},\;{\;D}^{\prime }=D+sign\left(N^{\prime }\right)N \end{array}$$

$$\begin{array}{c} \text{sign}(x)=\left\{ \begin{array}{cc} \text{\textbackslash{} \textbackslash{} 1}, & x>\text{0}\\ \text{\textbackslash{} \textbackslash{} 0}, & x=\text{0}\\ -\text{1}, & x<\text{0} \end{array}\right. \end{array}$$

$$\begin{array}{c} =\left\{ \begin{array}{cc} 1, & sign(N)=sign(\sigma )\\ 0, & otherwise \end{array}\right. \end{array}$$

**Proof:**

From Eq. (10), Eq. (3), and Eq. (11), $\dot{\sigma }$ can be obtained as follows:


$$\begin{array}{c} \dot{\sigma }=\frac{\vartheta }{{\text{2}e}_{m}}\text{sin}(\varphi -\theta )+\frac{\vartheta }{\text{2}e_{\theta_{m}}}\frac{\text{sin}\left(e_{\theta }\right)}{d}-\frac{\omega }{{\text{2}e}_{\theta_{m}}}=N\vartheta_{n}-D\omega_{n} \end{array}$$ (15)

Since


$$\begin{array}{c} N^{\prime }=\left\{ \begin{array}{cc} N+D\text{sign}(\sigma )\sqrt{\left|\sigma \right|}, & \text{sign}(N)=\text{sign}(\sigma )\\ D\text{sign}(\sigma )\sqrt{\left|\sigma \right|}, & \text{otherwise} \end{array}\right. \end{array}$$

Therefore, $\text{sign}(N^{\prime })=\text{sign }(\sigma )$. We have three cases as follows:

**Case 1:** |N|>D AND sign(N)=−sign(σ)

In this case, as shown in Eq. (14), $\omega_{n}=\text{0}$. Therefore, from Eq. (15), we obtain:


$$\begin{array}{c} \dot{\sigma }=N=-|N|\text{ sign}(\sigma ) \end{array}$$

**Case 2:** ${\text{ D}}^{\prime }=D+\text{sign}\left(N^{\prime }\right)*N=\text{0}$

This case occurs, when |N|=D AND sign(N)=−sign(σ). From Eq. (14), $\omega_{n}=\text{sign}(\sigma )$. Therefore,


$$\begin{array}{c} \dot{\sigma }=-D\text{sign }(\sigma ) \end{array}$$

**Case 3:** |N|<D or (|N|=D AND sign(N)=sign(σ))

In this case $\text{sign}\left(\omega_{n}\right)=\text{sign}(\sigma )$. One of the following cases may occur:

- In the case of $\left|\omega_{n}\right|>\text{1}$ (as a special case of $\left|\omega_{n}\right|>\text{1}$ when $D^{\prime }=\text{0}$), since |ω| cannot be greater than $\omega_{m}$, therefore $\omega_{n}$ must be clipped such that $\left|\omega_{n}\right|=\text{1}$. Consequently, the control signal is set to be $\omega_{n}=\text{sign}(\sigma )$, then from Eq. (3), Eq. (15) becomes:

$$\begin{array}{c} \dot{\sigma }=-D\text{sign }(\sigma ) \end{array}$$

- In the case of $\left|\omega_{n}\right|<\text{1}$, the control signal is $\omega_{n}=N^{\prime }/D^{\prime }$, as shown in Eq. (14). By substituting for $\omega_{n}$ in Eq. (15), we obtain:

$$\begin{array}{c} \dot{\sigma }=N\left(\text{1}-\left|\omega_{n}\right|\right)-D\omega_{n}=N-\omega_{n}\left(D+\text{sign}(\sigma )N\right) \end{array}$$

$$\begin{array}{c} =N\vartheta_{n}-\left(cN+D\text{sign}(\sigma )\sqrt{\left|\sigma \right|}\right) \end{array}$$

$$\begin{array}{c} \;=N\vartheta_{n}-\left(c|N|+D\sqrt{\left|\sigma \right|}\right)\text{sign}(\sigma ) \end{array}$$

Since $\vartheta_{n}\leq \text{1}\;,\;$therefore $\text{sign}\left(N\vartheta_{n}-\left(c|N|+D\sqrt{\left|\sigma \right|}\right)\text{sign}(\sigma )\right)=-$ sign(σ).

Let $V_{\sigma }$ be a Lyapunov function candidate, where $V_{\sigma }=(\text{1}/\text{2})\sigma^{\text{2}}$. Then, ${\dot{V}}_{\sigma }=\sigma \dot{\sigma }$. At t=0, the initial value of $V_{\sigma }$, where σ≠0, is ${V_{\sigma }}_{o}$. The time required to reach σ=0 ($V_{\sigma }=\text{0})\;$is $t_{f}$.

**In case 1**, ${\dot{V}}_{\sigma }=-|N||\sigma |=-|N|\sqrt{\text{2}V_{\sigma }}<-D\sqrt{\text{2}V_{\sigma }}$. $t_{f}$ is obtained by integrating $\int_{\text{0}}^{t_{f}}V_{\sigma }^{-\frac{\text{1}}{\text{2}}}{\dot{V}}_{\sigma }dt<\int_{\text{0}}^{t_{f}}-\sqrt{\text{2}}Ddt$. Thus, $t_{f}<\sqrt{\text{2}{V_{\sigma }}_{o}}\;/D$. Therefore, the system states are pushed to σ=0 in a finite time.

**In case 2**, ${\dot{V}}_{\sigma }=-D|\sigma |$. $t_{f}$ is obtained by integrating $\int_{\text{0}}^{t_{f}}V_{\sigma }^{-\frac{\text{1}}{\text{2}}}{\dot{V}}_{\sigma }dt=\int_{\text{0}}^{t_{f}}-\sqrt{\text{2}}Ddt$. Thus, $t_{f}=\sqrt{\text{2}{V_{\sigma }}_{o}}\;/D$. Thus, the system states are forced to σ=0 in a finite time.

**In case 3,** we have three cases as follows


$$\begin{array}{c} {\dot{V}}_{\sigma }=\left\{ \begin{array}{cc} -D\sqrt{\text{2}V_{\sigma }}\;, & \left|\omega_{n}\right|\geq \text{1}\\ -\left||N|\vartheta_{n}-\left(|N|+D\sqrt{\left|\sigma \right|}\right)\right|\sqrt{\text{2}V_{\sigma }}, & \left|\omega_{n}\right|<\text{1}\text{ and sign}(N)=\text{sign}(\sigma )\\ -\left||N|\vartheta_{n}+D\sqrt{\left|\sigma \right|}\right|\sqrt{\text{2}V_{\sigma }}, & \text{otherwise} \end{array}\right. \end{array}$$ (16)

In the first case, $t_{f}$ is obtained from $\int_{\text{0}}^{t_{f}}V_{\sigma }^{-\frac{\text{1}}{\text{2}}}{\dot{V}}_{\sigma }dt=\int_{\text{0}}^{t_{f}}-\sqrt{\text{2}}Ddt$ to be $t_{f}=\sqrt{\text{2}{V_{\sigma }}_{o}}\;/D$, which is a finite time. For the other two cases, in Eq. (16), ${\dot{V}}_{\sigma }\leq -{\text{2}}^{\frac{\text{3}}{\text{4}}}DV_{\sigma }^{\frac{\text{3}}{\text{4}}}$. Subsequently, by integrating $\int_{\text{0}}^{t_{f}}{V_{\sigma }^{-\frac{\text{3}}{\text{4}}}\dot{V}}_{\sigma }dt\leq \int_{\text{0}}^{t_{f}}-{\text{2}}^{\frac{\text{3}}{\text{4}}}Ddt$, we obtain $t_{f}\leq {\text{2}}^{\frac{\text{5}}{\text{4}}}{\left(V_{\sigma_{o}}\right)}^{\frac{\text{1}}{\text{4}}}/D$.

Therefore, at any instant σ≠0, the system states are always pushed back to σ=0in a finite time. Therefore, the system is maintained on the sliding surface σ=0. Thus, the proof is complete.

To eliminate the known chattering phenomenon associated with the sliding mode control technique, the sign() function is approximated as in Eq. (17) [17]:


$$\begin{array}{c} \text{sign}(x)\approx \frac{x}{|x|+\delta } \end{array}$$ (17)

where δ is a small positive scalar.

While the mechanical inertia of the DC motors inherently acts as a low-pass filter against high-frequency vibrations, applying raw discontinuous chattering directly to the motor driver circuits causes excessive wear and inductive heating. Therefore, the continuous approximation in Eq. (17) is utilized to smooth the control reference mathematically, protecting the driver electronics and preventing the excitation of unmodeled high-frequency electrical dynamics.

The proposed path-tracking approach is based on the kinematic model introduced in Eq. (10). In this approach, the states of the system are pushed to move on the sliding surface σ=0.Theorem 1 shows that tracking errors e and $e_{\theta }\;$approach zero as t→∞ on the sliding surface. Theorem 2 introduces a control law that guarantees the reachability of the sliding surface in finite time whenever σ≠0 and maintains the system there. In the following subsection, the effects of robot dynamics are investigated.

### 3.2. The effect of the system dynamics

So far, the effects of system dynamics have been ignored. If the axis of rotation passes through a point sufficiently close to the center of mass of the robot, and the linear and rotational velocities of the robot are not high (specifically limited to 70 cm/s and 10 rad/s, respectively), the Coriolis and centrifugal forces can be neglected. However, these motors are typically DC motors with negligible inductances [18]. The dynamics of the differential drive robot [18,19] coupled with the dynamics of the DC motors can be modeled as follows:


$$\begin{array}{c} J\omega^{\prime }=-B_{\omega }\omega +A_{\omega }{(V}_{R}-V_{L}) \end{array}$$

$$\begin{array}{c} {M\vartheta }^{\prime }=-B_{\vartheta }\vartheta +A_{\vartheta }{(V}_{R}+V_{L}) \end{array}$$ (18)

where M and J represent the total equivalent mass and the moment of inertia of the robot, wheels, and motors, respectively. $B_{\omega }$ and $B_{\vartheta }$ represent the friction and back EMF of the motors for rotational and linear motions, respectively. $V_{R}$ and $V_{L}$ are the voltages applied by the controller to the right and left motor, respectively. $A_{\omega }$ and $A_{\vartheta }$ are constants that depend on the physical parameters of the motors and the robot such as back EMF and torque constants, armature resistances, masses, moment of inertia, distance between wheels, and wheel radii. Therefore, owing to the dynamics in Eq. (18), the angular and linear velocities require time to reach the required values to maintain the motion on the sliding surface. At the current instant $t_{o}$, the normalized linear and rotational velocities are ${\vartheta_{o}}_{n}$ and ${\omega_{o}}_{n}$, respectively. The new linear and rotational velocities corresponding to the voltages newly applied to the motors by the controller are $\vartheta_{n}$ and $\omega_{n}$, respectively. Then the actual velocities $\omega_{n}^{\prime }$ and $\vartheta_{n}^{\prime }\;$at the instant $t_{o}+\tau$, where τ is the sampling period, can be calculated as follows:


$$\begin{array}{c} \omega_{n}^{\prime }=\omega_{n}\left(\text{1}-e^{-\mu_{r}\tau }\right)+\omega_{o_{n}}e^{-\mu_{r}\tau } \end{array}$$

$$\begin{array}{c} \vartheta_{n}^{\prime }=\vartheta_{n}\left(\text{1}-e^{-\mu_{l}\tau }\right)+\vartheta_{o_{n}}e^{-\mu_{l}\tau } \end{array}$$ (19)

where $\mu_{l}$ and $\mu_{r}$ represent the linear and rotational dynamics of the robot, respectively. By substituting $\omega_{n}^{\prime }$ and $\vartheta_{n}^{\prime }$ from Eq. (19) for $\omega_{n}$ and $\vartheta_{n}$ in Eq. (15), respectively. Thus, we obtain the actual $\dot{\sigma }$, as expressed in Eq. (20), which represents the rate of change in σ considering robot dynamics.


$$\begin{array}{c} \dot{\sigma }=N-D\omega_{n},\text{ where} \end{array}$$

$$\begin{array}{c} N=N\left(\text{1}-e^{-\mu_{l}\tau }\right)+N\vartheta_{o_{n}}e^{-\mu_{l}\tau }-D\omega_{o_{n}}e^{-\mu_{r}\tau } \end{array}$$

$$\begin{array}{c} D=D\left(\text{1}-e^{-\mu_{r}\tau }\right)+\text{sign}\left(\omega_{n}\right)N\left(\text{1}-e^{-\mu_{l}\tau }\right) \end{array}$$ (20)

Then the control law in this case can be defined as follows:


$$\begin{array}{c} \omega_{n}=\left\{ \begin{array}{cc} \text{0}, & \left|N|>\text{0}\text{ AND sign}(N)=-\text{sign}(\sigma \right)\\ \text{sign}(\sigma ), & D^{\prime }=\text{0}\\ \frac{N^{\prime }}{D^{\prime }}\;, & \text{otherwise} \end{array}\right. \end{array}$$

$$\begin{array}{c} N^{\prime }=cN+D\left(\text{1}-e^{-\mu_{min}\tau }\right)\text{ sign}(\sigma )\sqrt{\left|\sigma \right|} \end{array}$$

$$\begin{array}{c} D^{\prime }=\;D\left(\text{1}-e^{-\mu_{min}\tau }\right)+\text{sign}\left(N^{\prime }\right)N\left(\text{1}-e^{-\mu_{min}\tau }\right) \end{array}$$

$$\begin{array}{c} \mu_{min}\leq \text{min}\left(\mu_{r},\mu_{l}\right) \end{array}$$

$$\begin{array}{c} =\left\{ \begin{array}{cc} \text{1}, & \text{sign}\left(N\right)=\text{sign}(\sigma )\\ \text{0}, & \text{otherwise} \end{array}\right. \end{array}$$ (21)

The following theorem shows that the reachability of the sliding surface σ=0 is assured using the control law in Eq. (21).

**Theorem 3:** The Control law in Eq. (21) guarantees the reachability of the sliding surface in finite time and remains on it.

**Proof:**

From Eq. (21), we have the following cases:

**Case 1:** |N|>0 AND sign(N)=−sign(σ)

In this case, from Eq. (21) $\omega_{n}=\text{0}$. Consequently, in the next samples $\omega_{o_{n}}=\text{0}$ and $\vartheta_{o_{n}}=\text{1}$. Hence, $\dot{\sigma }=N$. Therefore, $\text{sign}\left(\dot{\sigma }\right)=\text{sign}(N)=-\text{sign}(\sigma ).$

**Case 2:** $D^{\prime }=\text{0}$.

This case occurs, when |N|=D AND sign(N)=−sign(σ). From Eq. (21), $\omega_{n}=\text{ sign}(\sigma )$. Consequently, in the next samples $\omega_{o_{n}}=\text{sign}(\sigma )$ and $\vartheta_{o_{n}}=\text{0}$. Hence, $\dot{\sigma }=\;-D\text{sign}(\sigma )e^{-\mu_{r}\tau }-D\text{sign}(\sigma )\left(\text{1}-e^{-\mu_{r}\tau }\right)=-D\text{sign}(\sigma )$, Therefore, $\text{sign}\left(\dot{\sigma }\right)=-\text{sign}(\sigma )$.

**Case 3:** $D^{\prime }>\text{0}$

In this case, no prior knowledge of the exact values of $\mu_{r}\text{ and }\mu_{l}$ is needed; only the lower bound $\mu_{min}$ is required. Because $\mu_{min}\leq \text{min}\left(\mu_{r},\mu_{l}\right)$, then D*/*$D^{\prime }\geq \text{1}$ and hence, substituting for $\omega_{n}$ from Eq. (21) into $\dot{\sigma }$ in Eq. (20) yields $\text{sign}\left(\dot{\sigma }\right)=-\text{sign}(\sigma )$.

Therefore, the system states are pushed to σ=0, where the tracking error approaches zero as time approaches infinity.

Let $V_{\sigma }$ be a Lyapunov function candidate, where $V_{\sigma }=\frac{\text{1}}{\text{2}}\sigma^{\text{2}}$. Then, ${\dot{V}}_{\sigma }=\sigma \dot{\sigma }$. At t=0, the initial value of $V_{\sigma }$, where σ≠0, is ${V_{\sigma }}_{o}$. The time required to reach σ=0 ($V_{\sigma }=\text{0})\;$is $t_{f}$.

**In case 1:** $\dot{V}=-|N||\sigma |\leq -\epsilon |\sigma |=-\epsilon \sqrt{\text{2}V}$, where 0<ϵ≤|N|. Thus, $t_{f}\leq \sqrt{\text{2}{V_{\sigma }}_{o}}\;/\epsilon$. Therefore, the system states are pushed to σ=0 in a finite time.

**In case 2:** $\dot{V}=-D|\sigma |=-D\sqrt{\text{2}V}$. Therefore, $t_{f}=\sqrt{\text{2}{V_{\sigma }}_{o}}\;/D$. Thus, the system states are forced to σ=0 in a finite time.

**In case 3:** as shown in theorem 2, when $\left|\omega_{n}\right|>\text{1}$ it is clipped to be $\omega_{n}=\text{sign}(\sigma )$. Thus, we have the following cases:


$$\begin{array}{c} {\dot{V}}_{\sigma }=\left\{ \begin{array}{cc} -D\sqrt{\text{2}V_{\sigma }}\;, & \left|\omega_{n}\right|\geq \text{1}\\ -\left|\left|N\right|-\left(\left|N\right|+D\left(\text{1}-e^{-\mu_{min}\tau }\right)\sqrt{\left|\sigma \right|}\right)\frac{D}{D^{\prime }}\right|\sqrt{\text{2}V_{\sigma }}, & \left|\omega_{n}\right|<\text{1}\text{ and sign}\left(N\right)=\text{sign}(\sigma )\\ -\left|\left(\left|N\right|+D\left(\text{1}-e^{-\mu_{min}\tau }\right)\sqrt{\left|\sigma \right|}\right)\frac{D}{D^{\prime }}\right|\sqrt{\text{2}V_{\sigma }}, & \text{otherwise} \end{array}\right. \end{array}$$

Similar to theorem 2, in the first case, $t_{f}=\sqrt{\text{2}{V_{\sigma }}_{o}}\;/D$, which is a finite time. For the other two cases, ${\dot{V}}_{\sigma }\leq -{\text{2}}^{\frac{\text{3}}{\text{4}}}DV_{\sigma }^{\frac{\text{3}}{\text{4}}}$. Subsequently, by integration $t_{f}\leq {\text{2}}^{\frac{\text{5}}{\text{4}}}{\left(V_{\sigma_{o}}\right)}^{\frac{\text{1}}{\text{4}}}/D$.

Therefore, at any instant σ≠0, the system states are always pushed back to σ=0 in a finite time. Therefore, the system is maintained on the sliding surface σ=0. Thus, the proof is complete.

### 3.3. Path handling

The desired path is defined by a set of waypoints as shown in Fig 1. These waypoints are connected by straight segments. These waypoints are targeted sequentially. The path handler module shown in Fig 3 calculates $\varphi ,\;e,\;e_{\theta },$ and φ−θ. The angle φ between the segment connecting the targeted waypoint $P_{D}^{\left(k\right)}$ and the previous waypoint $P_{D}^{\left(k-\text{1}\right)}$ is calculated as follows:

**Fig 3:**
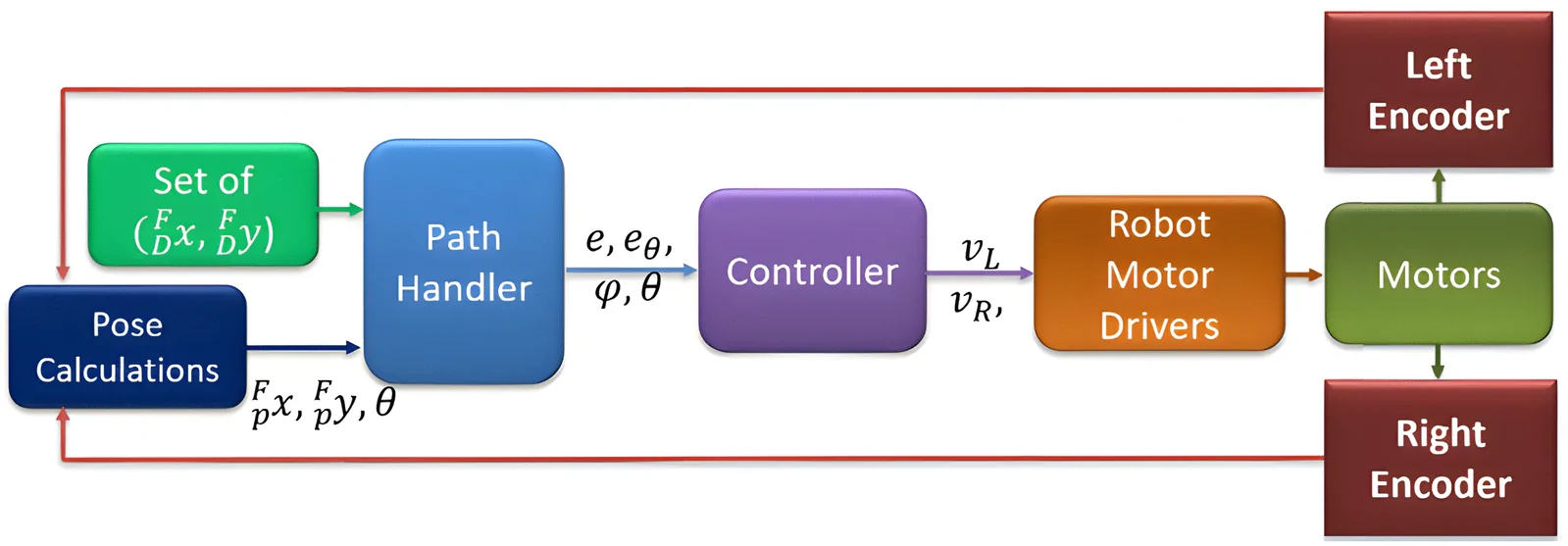
Control system.


$$\begin{array}{c} \varphi =\left\{ \begin{array}{c} {\text{tan}}^{-\text{1}}\left(\frac{DFy^{\left(k\right)}{-}_{D}^{F}y^{\left(k-\text{1}\right)}}{DFx^{\left(k\right)}{-}_{D}^{F}x^{\left(k-\text{1}\right)}}\right),\;k>\text{1}\\ {\text{tan}}^{-\text{1}}\left(\frac{DFy^{\left(k\right)}{-}_{p}^{F}y}{DFx^{\left(k\right)}{-}_{p}^{F}x}\right),\text{ otherwise} \end{array}\right. \end{array}$$

The tracking errors $e,\text{ and }e_{\theta }$ are calculated using Eq. (5) and Eq. (6), respectively. The path handler changes the targeted waypoint using the following algorithm until the last waypoint $P_{D}^{\left(k_{m}\right)}$ is reached, where the index of the last waypoint is $k_{m}$.

1. At the beginning, the waypoint index k is initiated by setting k=1.
2. The distance d is calculated by $d=\sqrt{{\left(DF{x^{\left(k\right)}-}_{p}^{F}x\right)}^{\text{2}}+{\left(DF{y^{\left(k\right)}-}_{p}^{F}y\right)}^{\text{2}}}$.
3. If (k>1) then
  If ($d\leq d_{tol}$ AND $k<k_{m}$) then

k=k+1

  End
  Elseif ($d\leq d_{tol})$ then

k=k+1

  End
4. Calculates $\varphi ,\;e,\;e_{\theta },$ andφ−θ

In the next subsection, the entire control system is described.

### 3.4. Control System

The complete control system is illustrated in Fig 3. The control algorithm can be summarized as follows:

1. A set of waypoints on the desired path is determined.
2. Pose Calculation:

a. The speeds of the left and right wheels are measured to calculate the actual angular velocity ω and linear ϑ from Eq. (1).
b. The current pose of the robot is numerically calculated using the odometry equations in Eq. (22), which are required for the microcontroller to calculate the real-time pose from encoder data.

$$\begin{array}{c} \theta (n)=\left(\frac{s_{r}C_{r}-s_{l}C_{l}}{w}\right)\rho +\theta (n-\text{1}) \end{array}$$

$$\begin{array}{c} pFx(n)=\left(\frac{s_{r}C_{r}+s_{l}C_{l}}{\text{2}}\right)\rho \text{cos}\left(\frac{\theta (n)+\theta (n-\text{1})}{\text{2}}\right){+}_{p}^{F}x(n-\text{1}) \end{array}$$

$$\begin{array}{c} pFy(n)=\left(\frac{s_{r}C_{r}+s_{l}C_{l}}{\text{2}}\right)\;\rho \text{ sin}\left(\frac{\theta (n)+\theta (n-\text{1})}{\text{2}}\right){+}_{p}^{F}y(n-\text{1}) \end{array}$$ (22)

where $s_{r}$ and $s_{l}$ are the direction of the right wheel and left wheel, respectively. The direction equals 1 for forward and −1 for reverse. $C_{r}$ and $C_{l}$ are the counts of the right and left encoders, respectively, during the sampling period. The current updated pose is θ(n)*,* $\backslash {(}_{p}^{F}x(n)$*,* and $\backslash {(}_{p}^{F}y(n).$ Whereas θ(n−1)*,* $\backslash {(}_{p}^{F}x(n-\text{1})$*,* and $\backslash {(}_{p}^{F}y(n-\text{1})$ are the previous pose. The linear displacement corresponding to each pulse is ρ, which equals the reciprocal of the encoder resolution.

3. Path Handler:
  a. Receives a set of the desired waypoints.
  b. Determines the current waypoint $P_{D}^{\left(k\right)}$and calculates the associated errors and angles.
  c. Calculates $\varphi ,\;e,\;e_{\theta },$ and φ−θ.
  d. Sends $e,\;e_{\theta }$, and φ−θ to the controller.
4. Controller:
  a. Calculates the angular velocity from:
    i. In the case of ignored robot dynamics, Eq. (14) is used.
    ii. When considering robot dynamics, Eq. (21) is used.
  b. Calculates the linear velocity ϑ from Eq. (3).
  c. The desired right $\vartheta_{R}$ and left $\vartheta_{L}$ velocities are calculated using Eq. (1).

In the next section, simulations are conducted to show the effect of the controller parameters and to compare the proposed technique with that introduced in [15]. Subsequently, the proposed technique was tested using a differential robot.

## 4. Simulation and experimental tests

This section is divided into two subsections. The first subsection 4.1 presents simulations conducted to demonstrate the effects of the controller parameters. Then, the proposed controller is compared with the path-tracking controller in [15] (hereafter referred to as the benchmark controller), which is a modified version of the control approach introduced in [2] (the control approach in [2] is widely used in many recent path-tracking control systems such as [4–6]). In subsection 4.2, the proposed controller is applied to an actual differential drive robot.

### 4.1. Simulations

Simulations were conducted to demonstrate the effects of the controller parameters and to compare the proposed controller with the benchmark controller assuming that the robot dynamics were ignored. Consequently, the control law of the proposed controller expressed by Eq. (14) was used.

Three simulations were performed to illustrate the effects of the controller parameters $e_{m},\;{e_{\theta }}_{m}and\;\delta$. The values of $\vartheta_{m},\omega_{m},\;$the sampling period, and $d_{tol}$ were 70 cm/s, 10 rad/s, $\text{2}\times {\text{1}\text{0}}^{-\text{3}}s$ and 1 cm, respectively. First, ${e_{\theta }}_{m}and\;\delta$ were set as π/18 rad and 0.01, respectively. The results obtained using the two values of $e_{m}$(1 cm and 10 cm) are depicted in Fig 4 and Fig 5. Second, $e_{m}\;$and δ were set as 1 cm and 0.01, respectively. Two values of ${e_{\theta }}_{m}$ (π/18 rad and π/2 rad) were used to demonstrate the effect of this parameter, as shown in Fig 6 and Fig 7. Third, $e_{m}\;and\;{e_{\theta }}_{m}$ were set as 1 cm and π/18 rad, respectively. The results of setting the parameter δ to two different values (0.01 and 0) are illustrated in Fig 8 and Fig 9.

**Fig 4:**
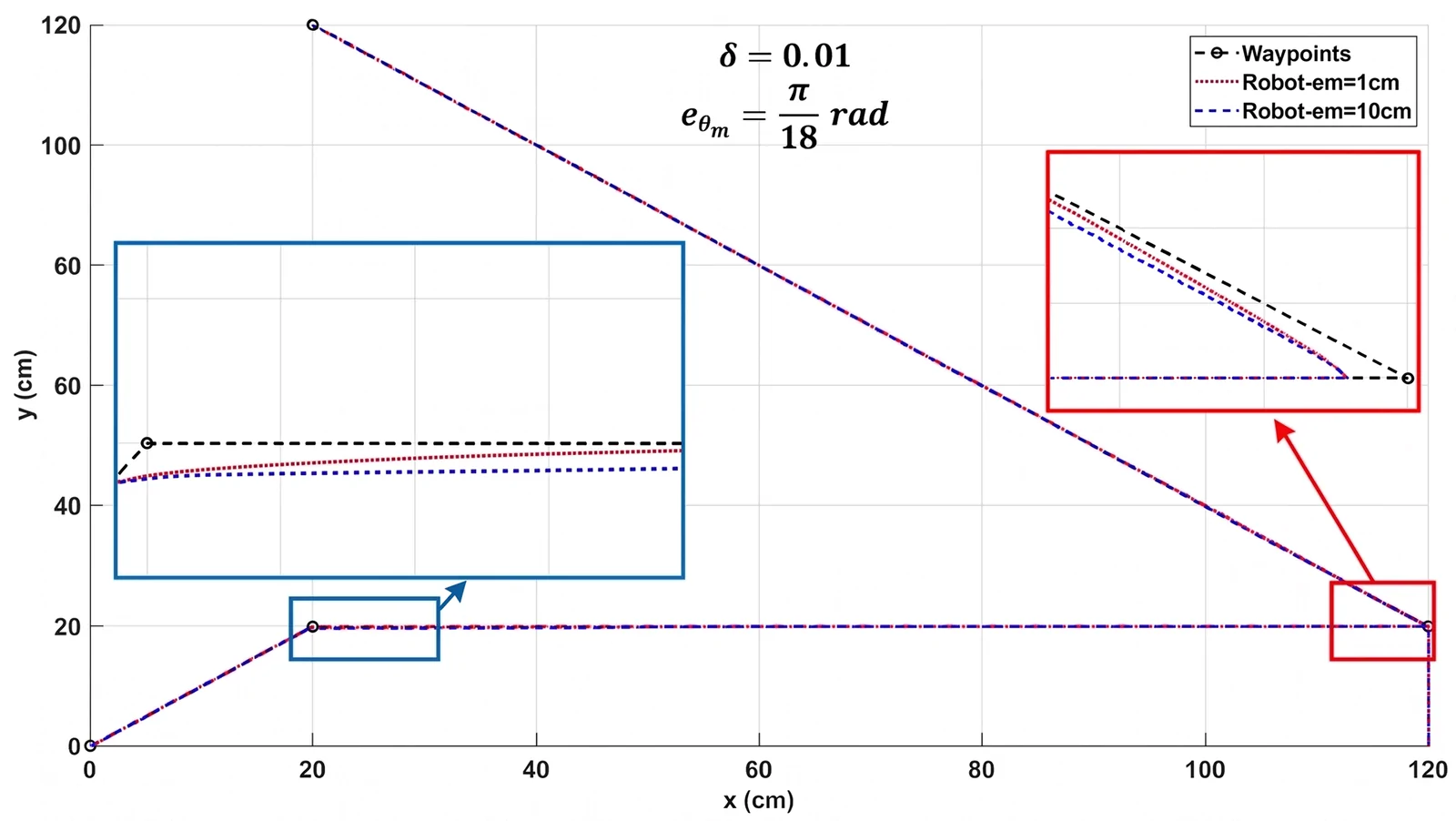
The effect of $e_{m}$ on the controller performance.

**Fig 5:**
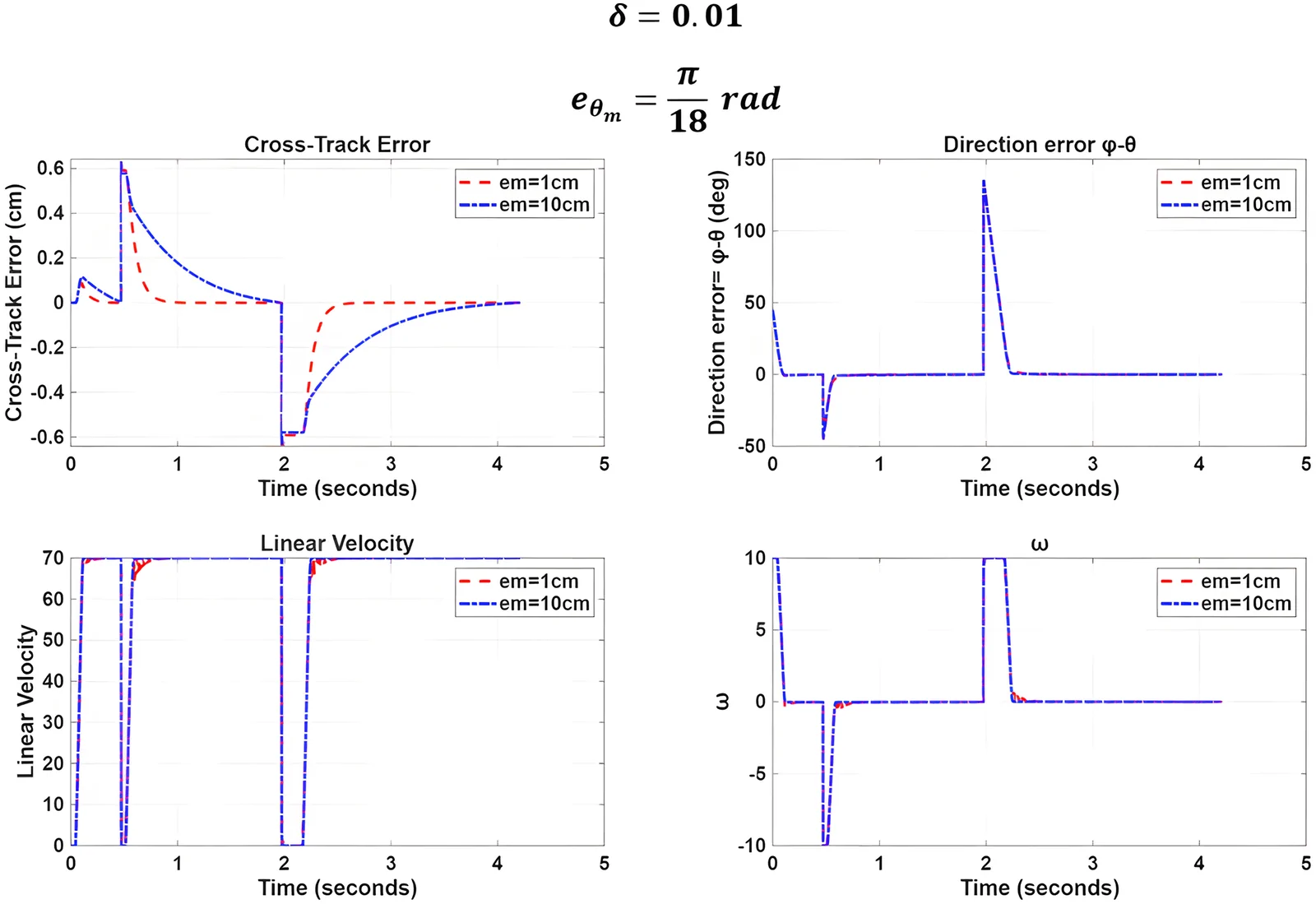
The effect of $e_{m}$ on cross-track error, linear velocity, direction error, and ω.

**Fig 6:**
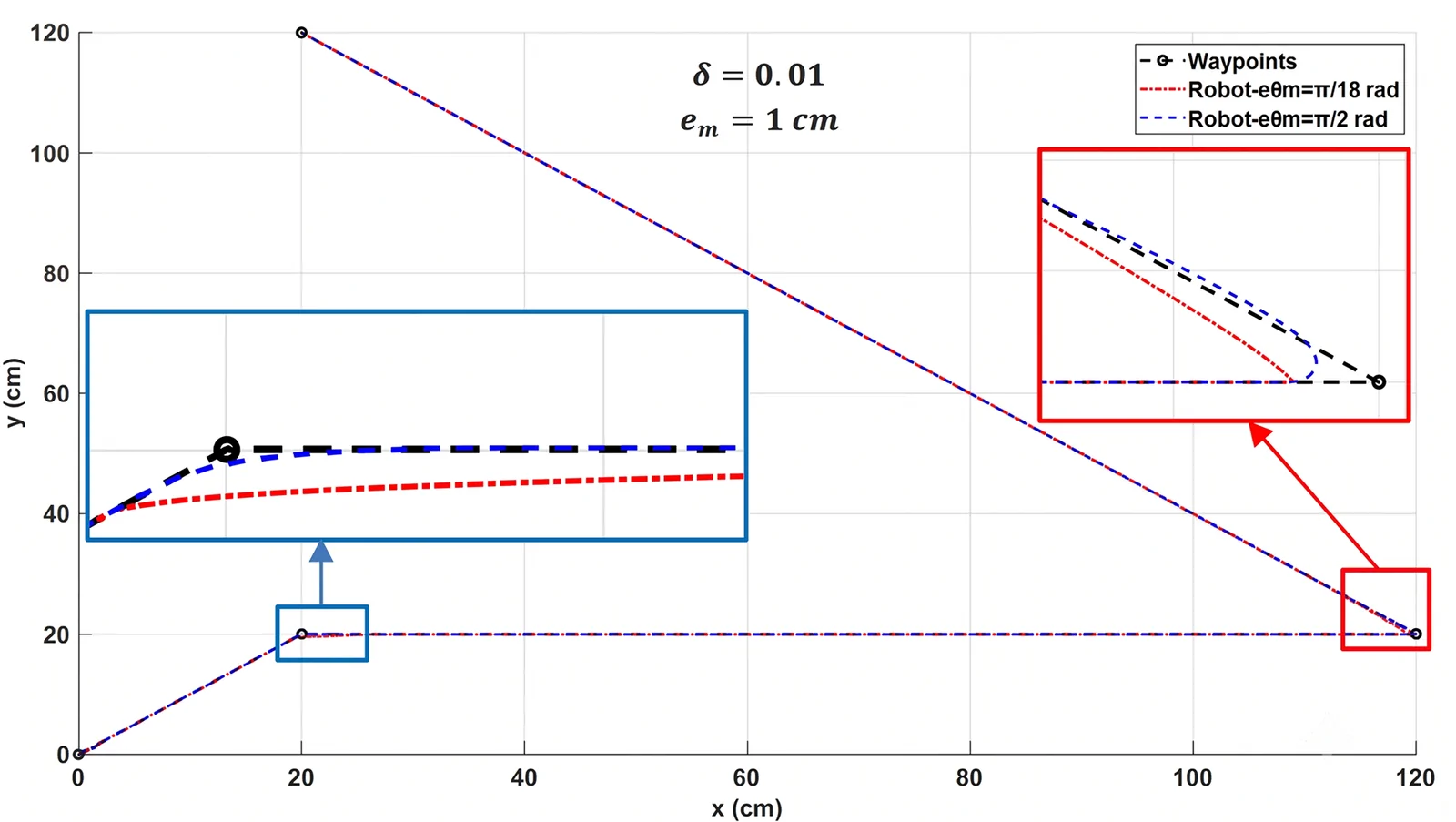
The effect of $e_{\theta_{m}}\;$ on the controller performance.

**Fig 7:**
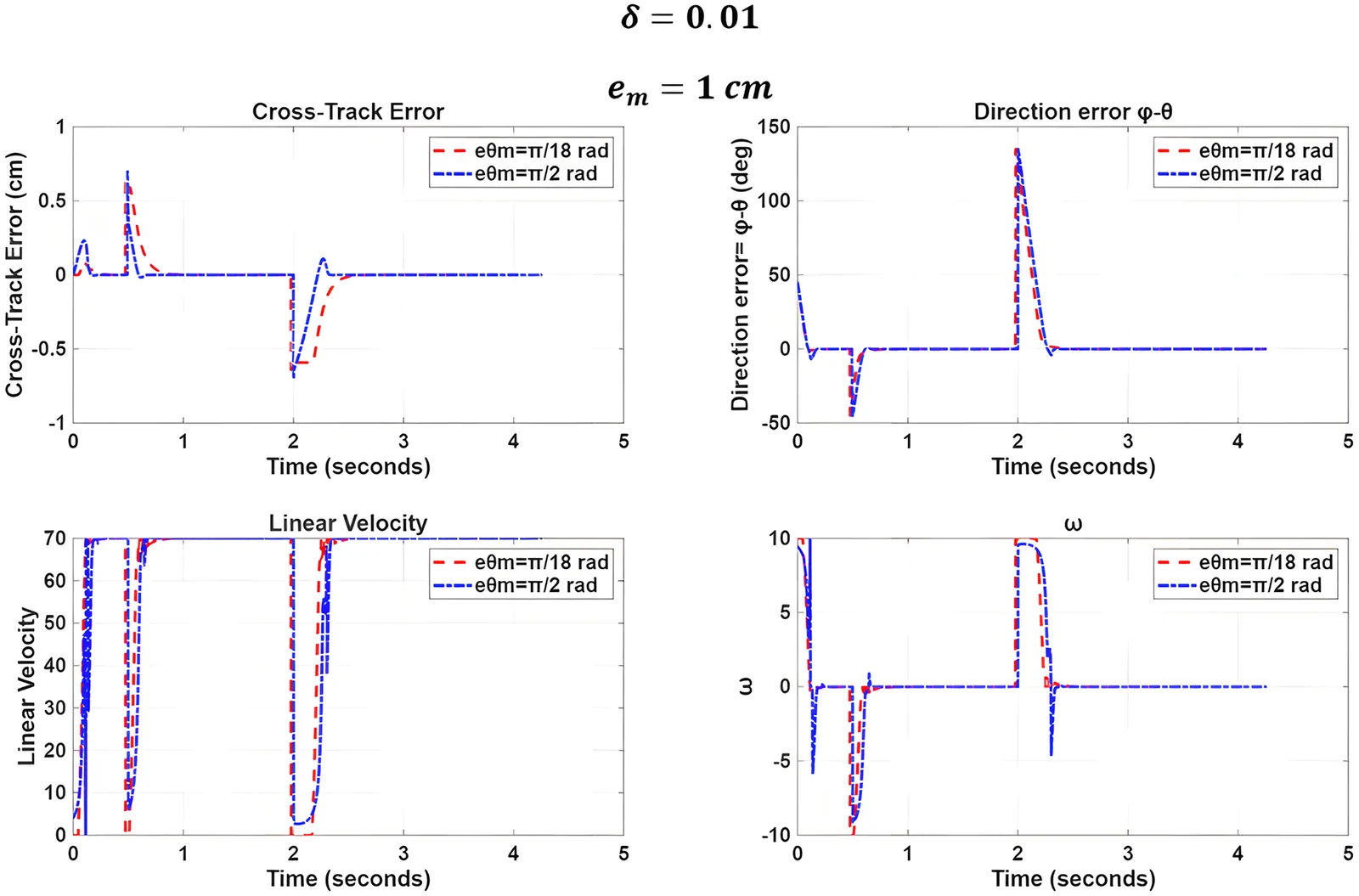
The effect of $e_{\theta_{m}}\;$ on cross-track error, linear velocity, direction error, and ω.

**Fig 8:**
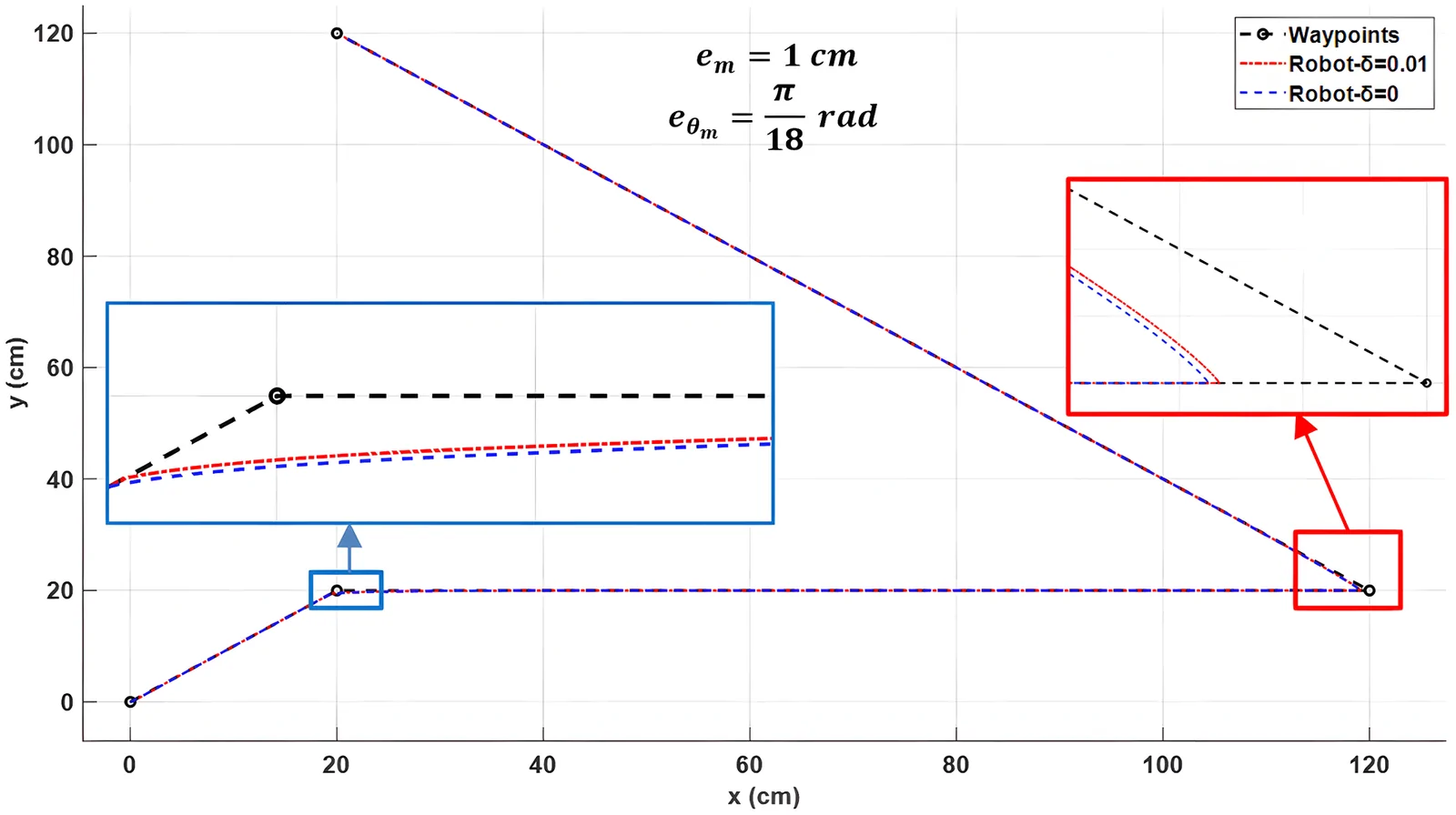
The effect of δ on the controller performance.

**Fig 9:**
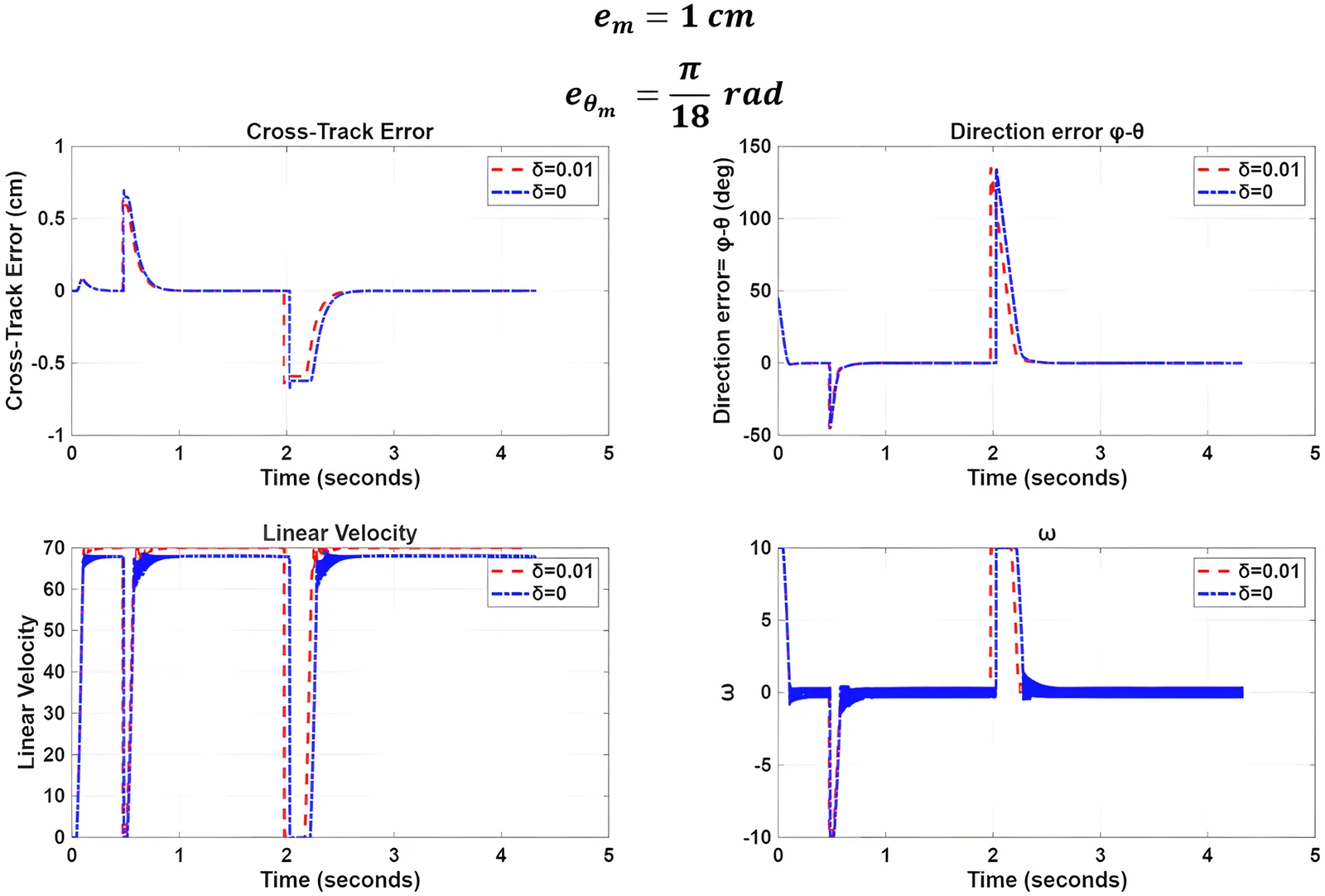
The effect of δ on cross-track error, linear velocity, direction error, and ω.

From the simulation results, increasing $e_{m}\;$reduces the weight of the cross-track error relative to the angular error, causing the cross-track error to take longer to vanish, as shown in Fig 4. In contrast, a larger ${e_{\theta }}_{m}$ decreases the impact of the angular error, leading to a faster disappearance of the cross-track error with an overshoot, as depicted in Fig 6. Moreover, from Eq. (17), sign() is exact when δ=0 that induces chattering in the angular velocity control signal as illustrated in Fig 9. Due to fluctuations in the control signal, the linear velocity is slower than in the case of δ=0.01, which produces a smoother control signal.

Subsequently, the proposed controller was compared with the benchmark controller. The robot was initially positioned at the first waypoint: $\backslash {(}_{p}^{F}x=\text{0}$, $\backslash {(}_{p}^{F}y=\text{0}$, and θ=0 rad. The parameters of the benchmark controller $k_{\text{1}}\;and\;k_{\text{2}}$ are set to 1.51 and 45.84, respectively, as calculated in [15]. The reference linear and angular velocities of the benchmark controller are 70 cm/s and 0 rad/s, respectively. On the other hand, $\vartheta_{m},\omega_{m},\;e_{m},\;{e_{\theta }}_{m}and\;\delta$ of the proposed controller are 70 cm/s, 10 rad/s, 1 cm, π/18 rad, and 0.01, respectively. For both the benchmark and proposed controllers, the sampling period and parameter $d_{tol}$ were $\text{2}\times {\text{1}\text{0}}^{-\text{3}}s$ and 1 cm, respectively.

As shown in Fig 10–Fig 21, a comparison was made using three paths defined by four waypoints. The robot was required to perform two turns of π/4rad at the 1^st^ and 2^nd^ waypoints. At the 3^rd^ waypoint, the robot performed turning angles of π/4,π/2, and 3π/4 rad as depicted in Fig 10, Fig 14, and Fig 18, respectively. As illustrated in the figures of the three paths, the path of the robot with the proposed controller is very tight compared with that of the benchmark controller at turns with directional discontinuity and/or sharp turns. The proposed controller follows the desired straight segments between waypoints more accurately than the benchmark controller does. In Table 1, the average of the cross-track errors of both the proposed and the benchmark controller are presented. This average was calculated using Eq. (23), where τ is the sampling period. As shown in the table, the proposed controller significantly reduced the average cross-track error. The maximum absolute cross-track error achieved by the proposed controller was much smaller than that achieved by the benchmark controller, as shown in Table 2. Therefore, the proposed controller outperforms the benchmark controller in terms of maneuvers with directional discontinuities and/or sharp turns.

**Table 1:** Average Cross-Track error.

Type of waypoint trajectory	Average of error (cross track error) (cm)		
**Proposed Controller**	**Controller in [15]**	**Percentage Improvement (percentage of error reduction)**
**A path with an angle of** $\frac{\pi }{4}$ **rad**	0.0436	0.2877	84.8%
**A path with an angle of** $\frac{\pi }{2}$ **rad**	0.0806	0.4722	82.9%
**A path with an angle of** $\frac{3\pi }{4}$ **rad**	0.0654	0.456	85.7%

**Table 2:** Maximum Cross-Track error.

Type of waypoint trajectory	Maximum absolute cross-track error (cm)		
**Proposed Controller**	**Controller in [15]**	**Percentage Improvement (percentage of error reduction)**
**A path with an angle of** $\frac{\pi }{4}$ **rad**	0.6414	1.7062	62.4%
**A path with an angle of** $\frac{\pi }{2}$ **rad**	0.9070	2.5079	63.8%
**A path with an angle of** $\frac{3\pi }{4}$ **rad**	0.6414	2.7444	76.6%

**Fig 10:**
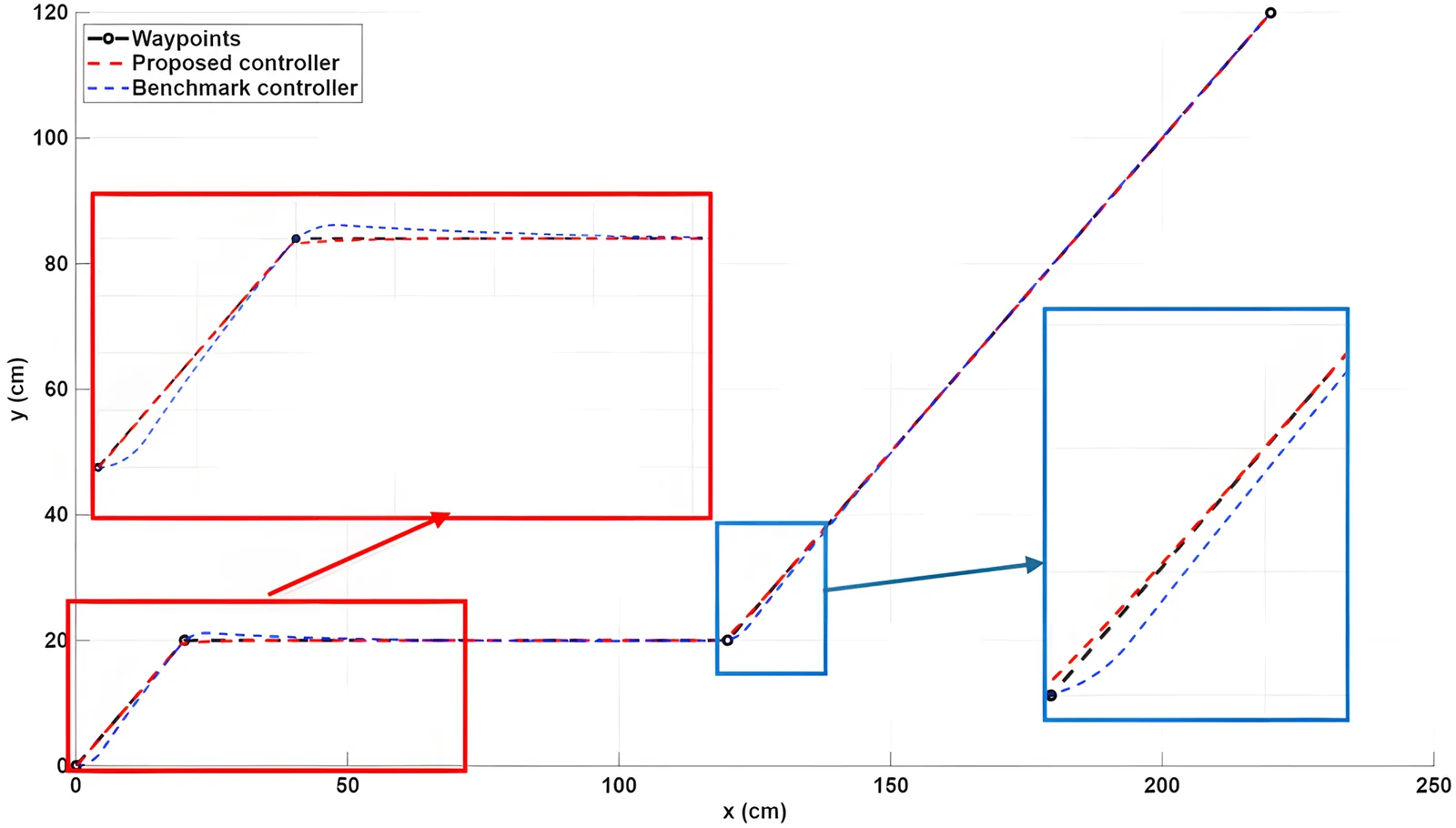
A path with an angle of π/4 rad.

**Fig 11:**
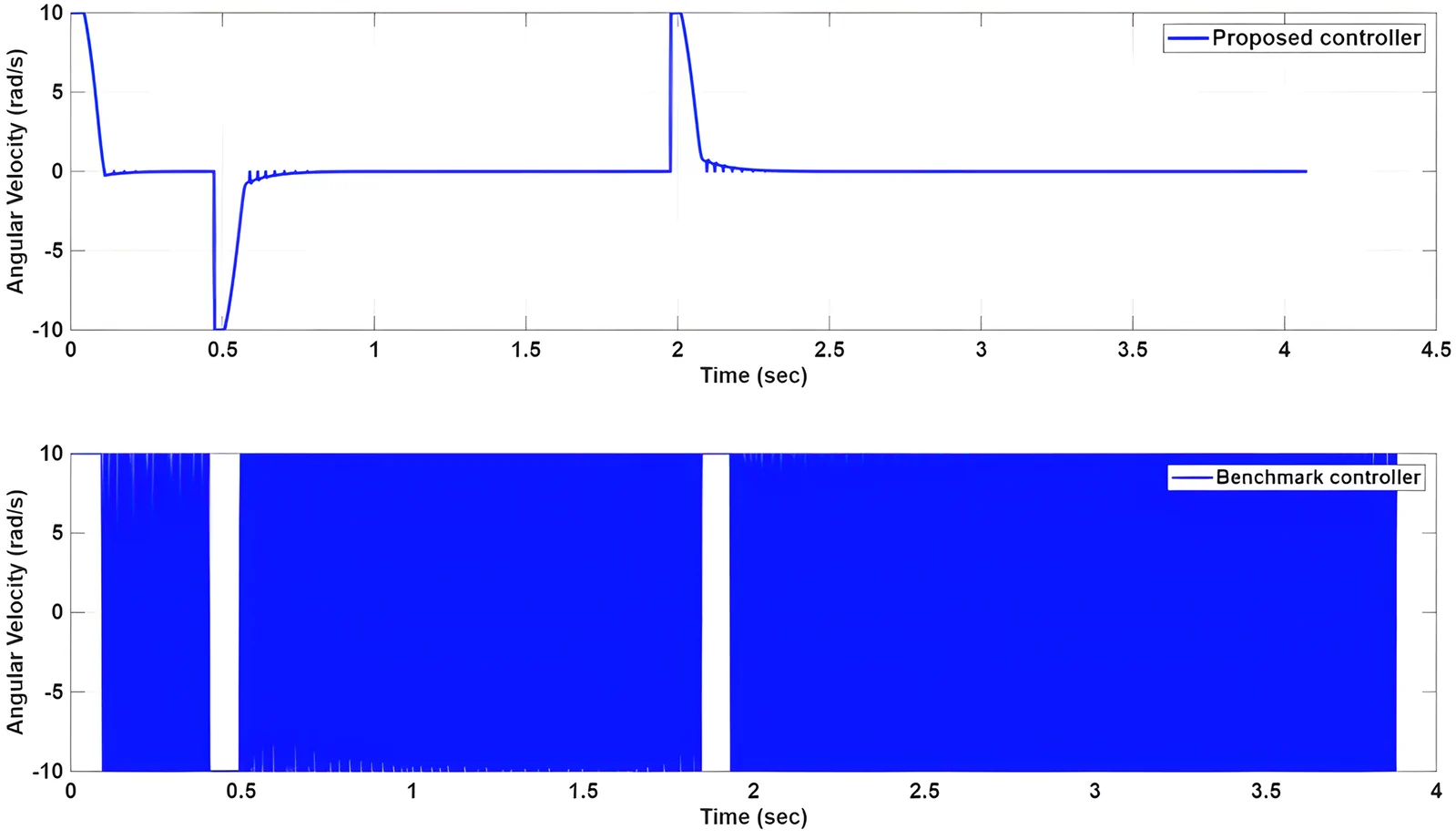
The angular velocity on the path with an angle of π/4 rad.

**Fig 12:**
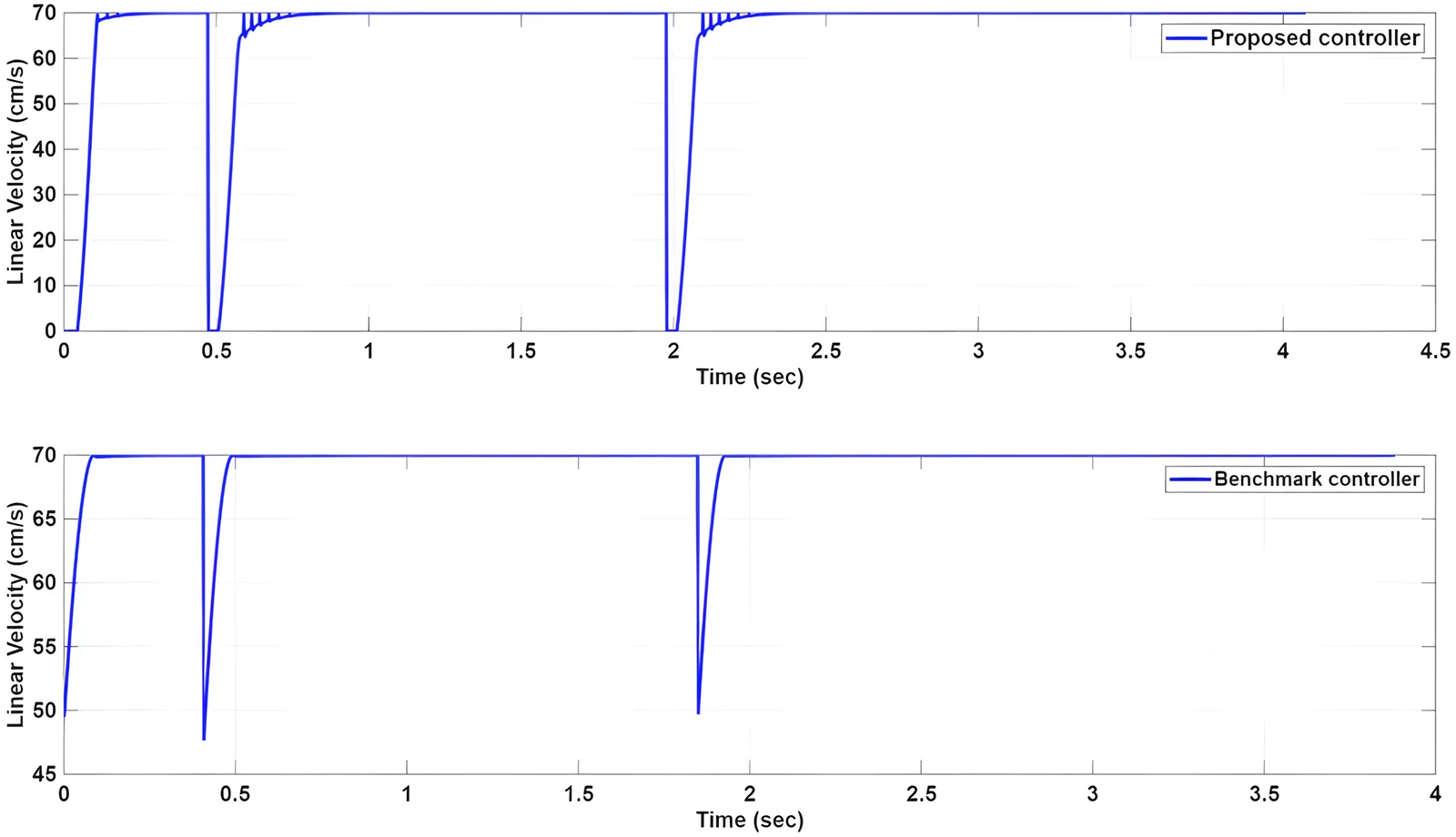
The linear velocity on the path with an angle of π/4 rad.

**Fig 13:**
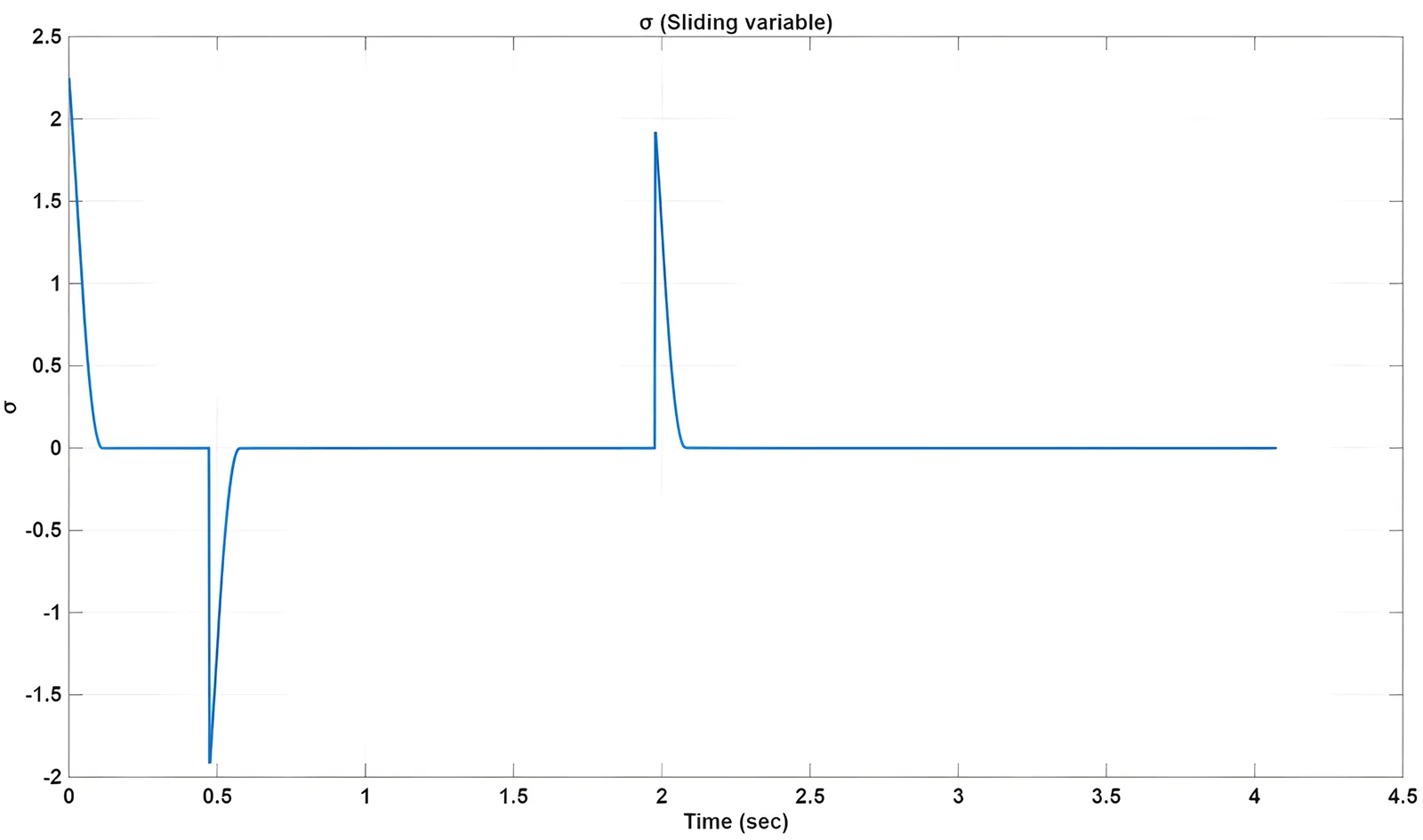
The sliding variable σ on the path with an angle of π/4 rad.

**Fig 14:**
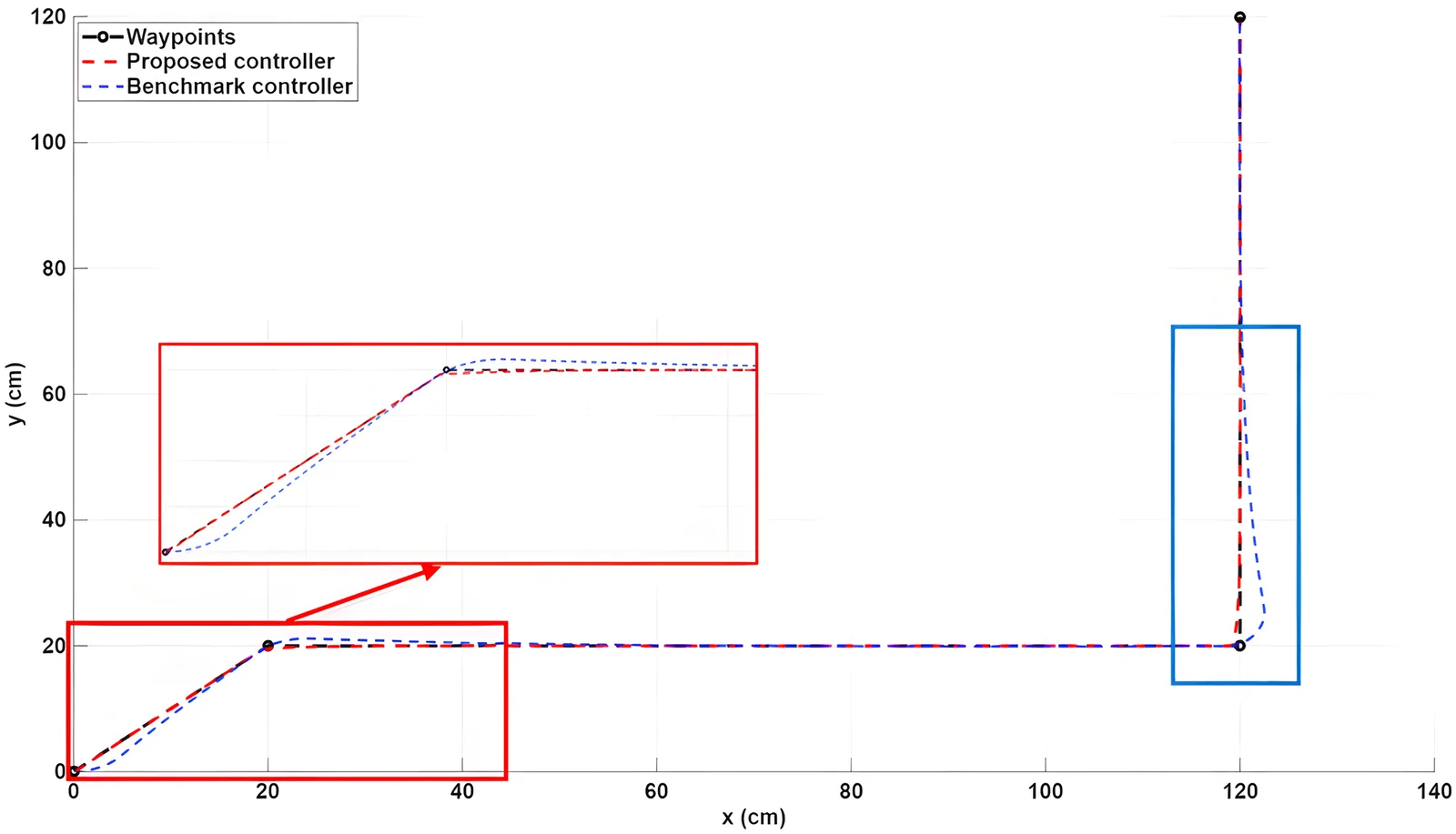
A path with an angle of π/2 rad.

**Fig 15:**
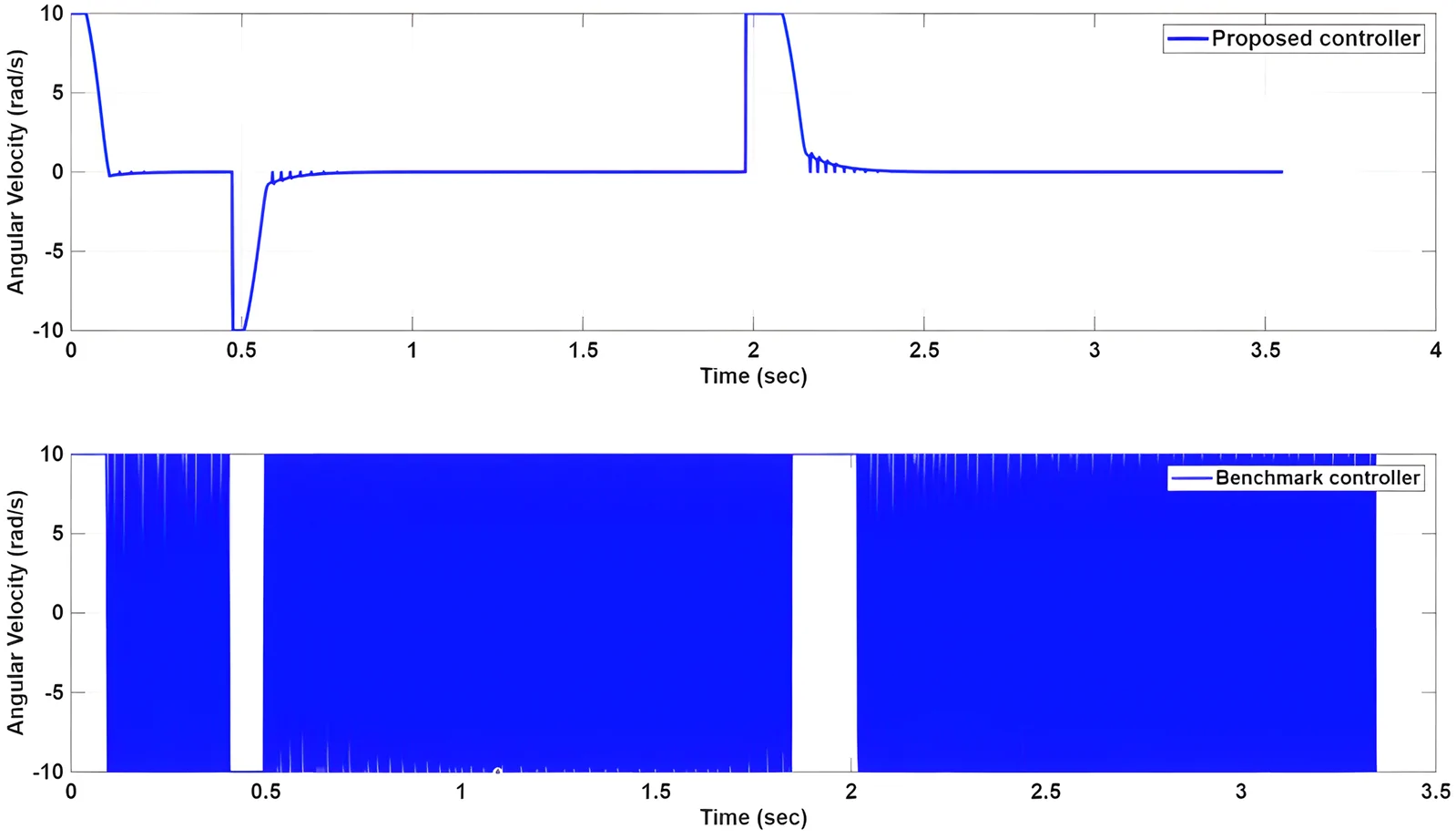
The angular velocity on the path with an angle of π/2 rad.

**Fig 16:**
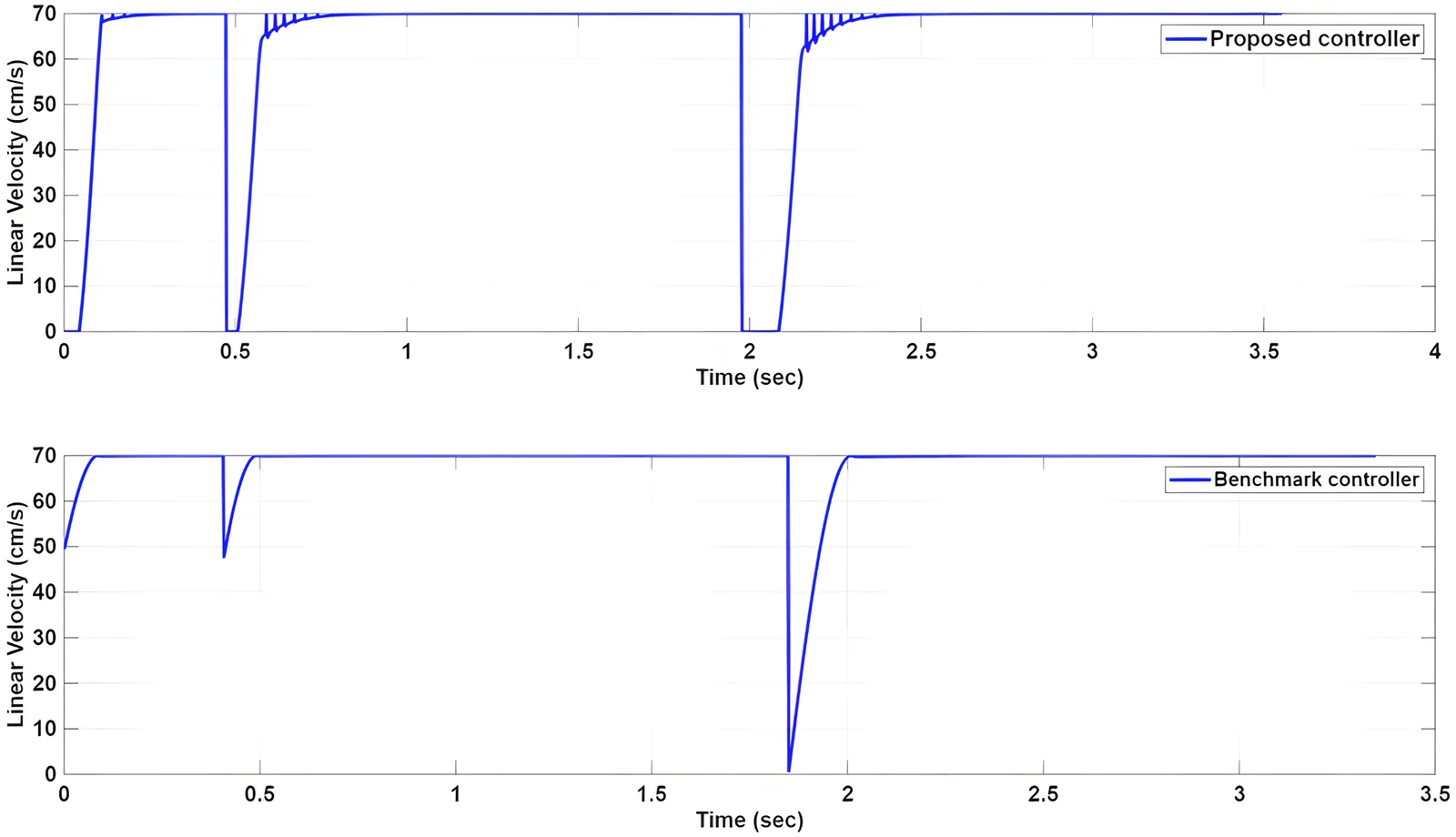
The linear velocity on the path with an angle of π/2 rad.

**Fig 17:**
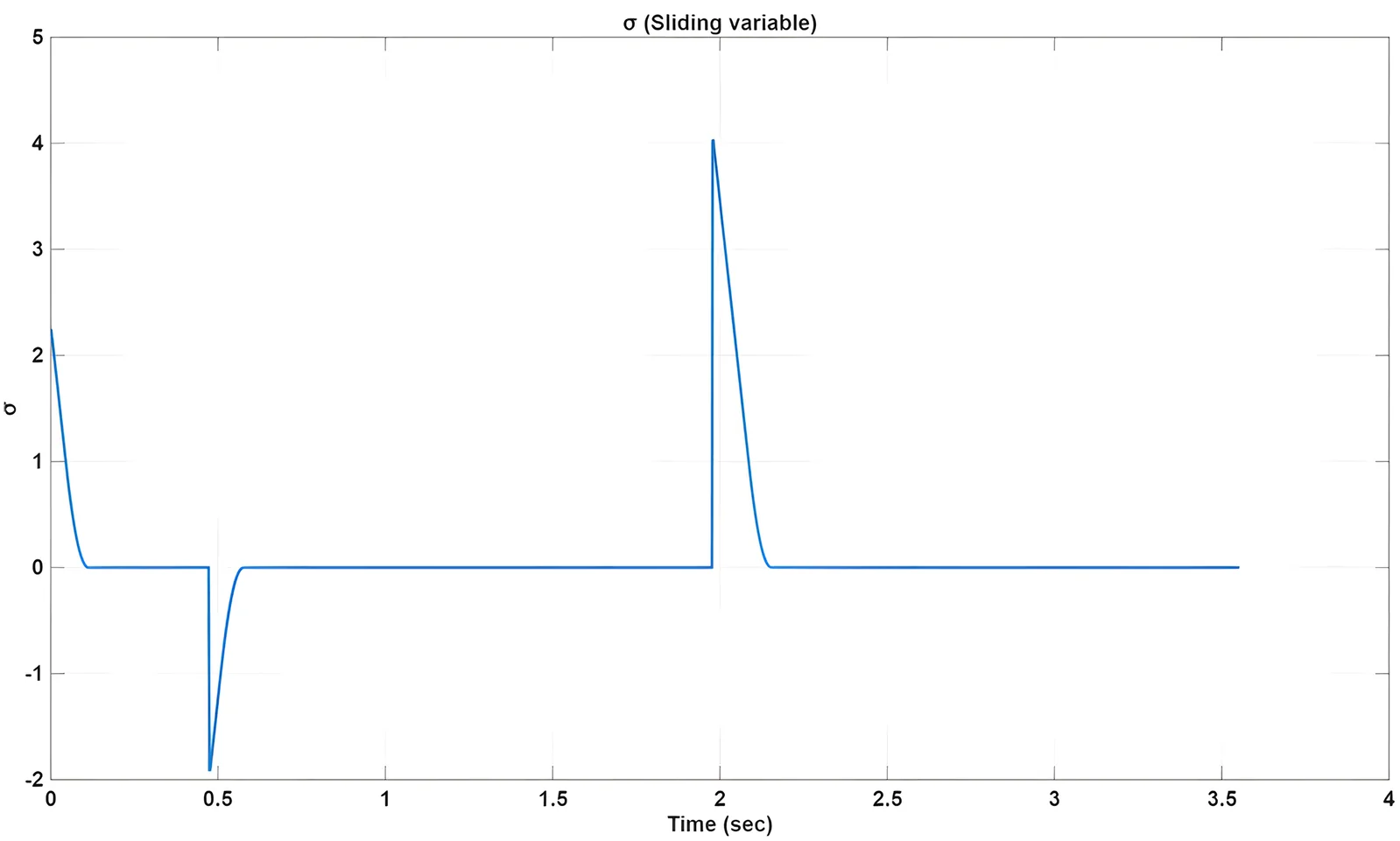
The sliding variable σ on the path with an angle of π/2 rad.

**Fig 18:**
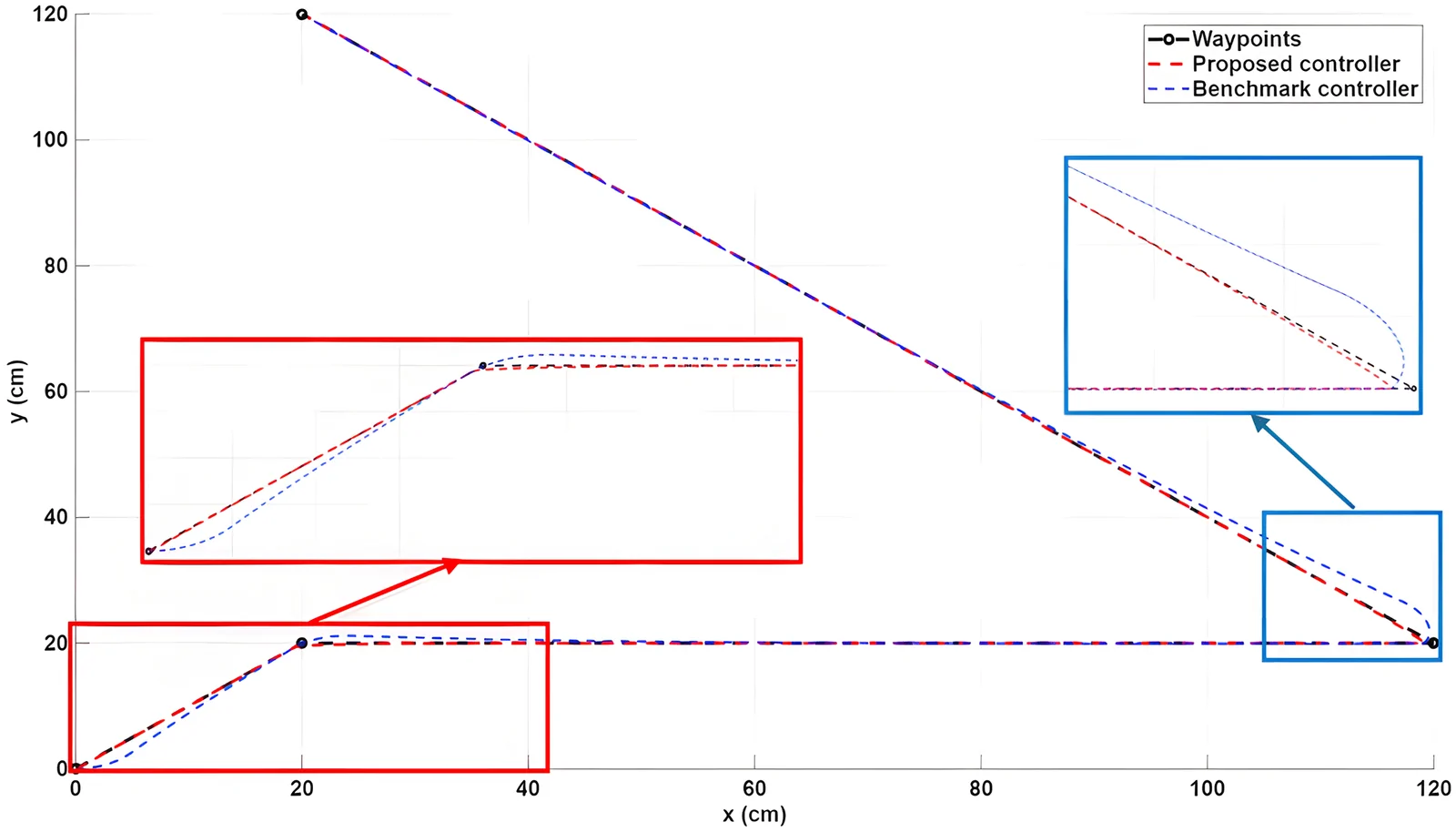
A path with an angle of 3π/4 rad.

**Fig 19:**
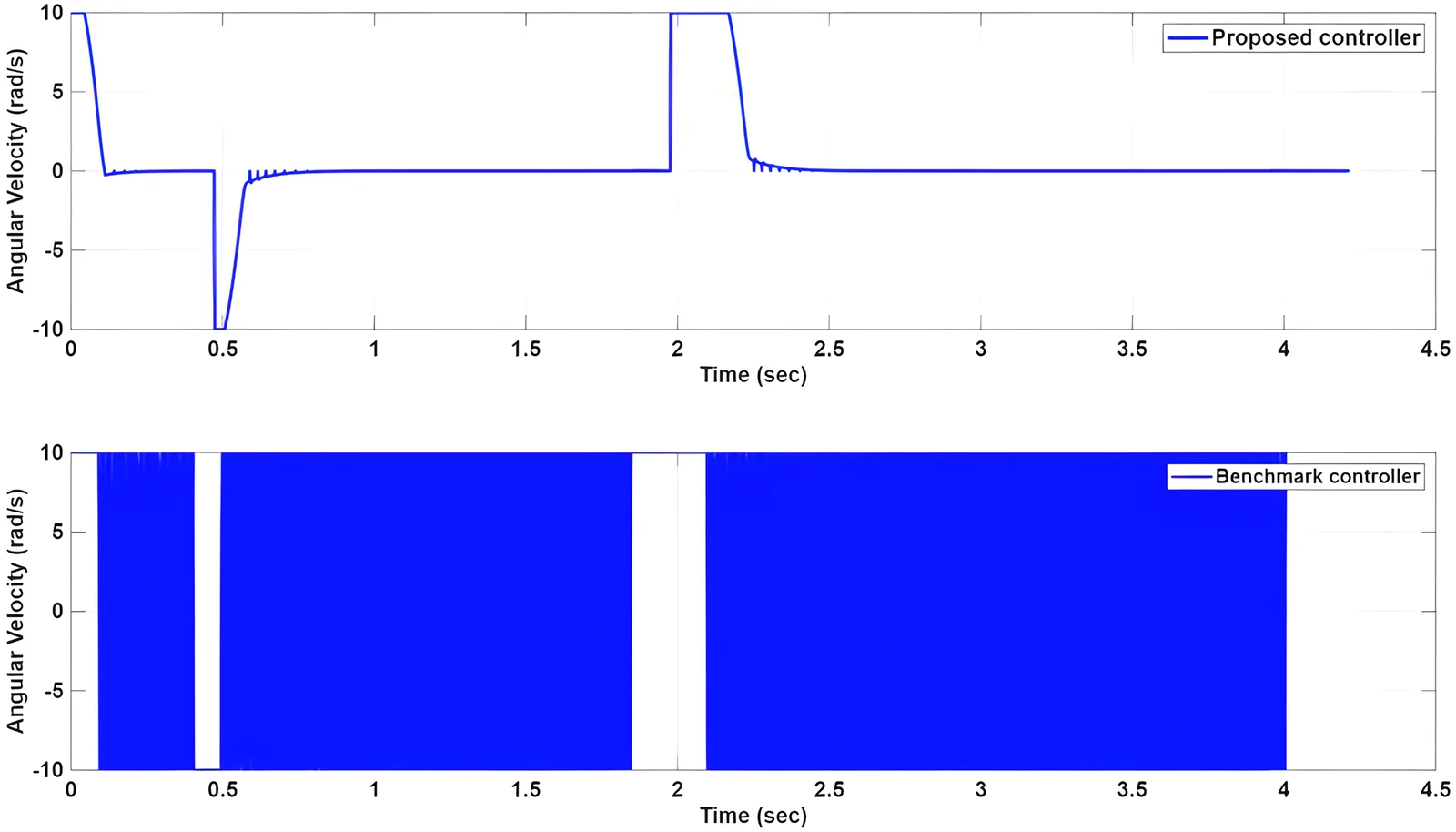
The angular velocity on the path with an angle of 3π/4 rad.

**Fig 20:**
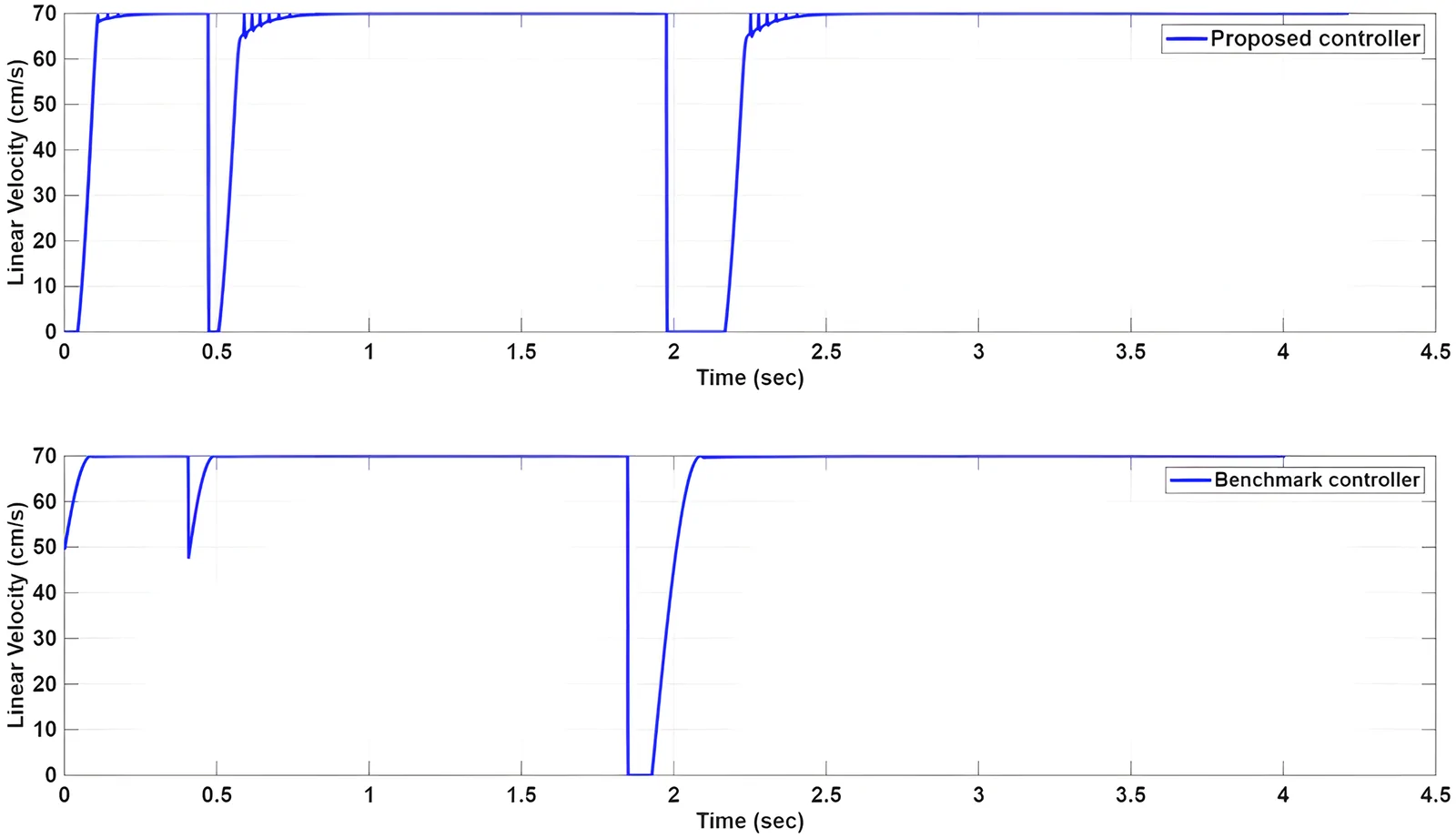
The linear velocity on the path with an angle of 3π/4 rad.

**Fig 21:**
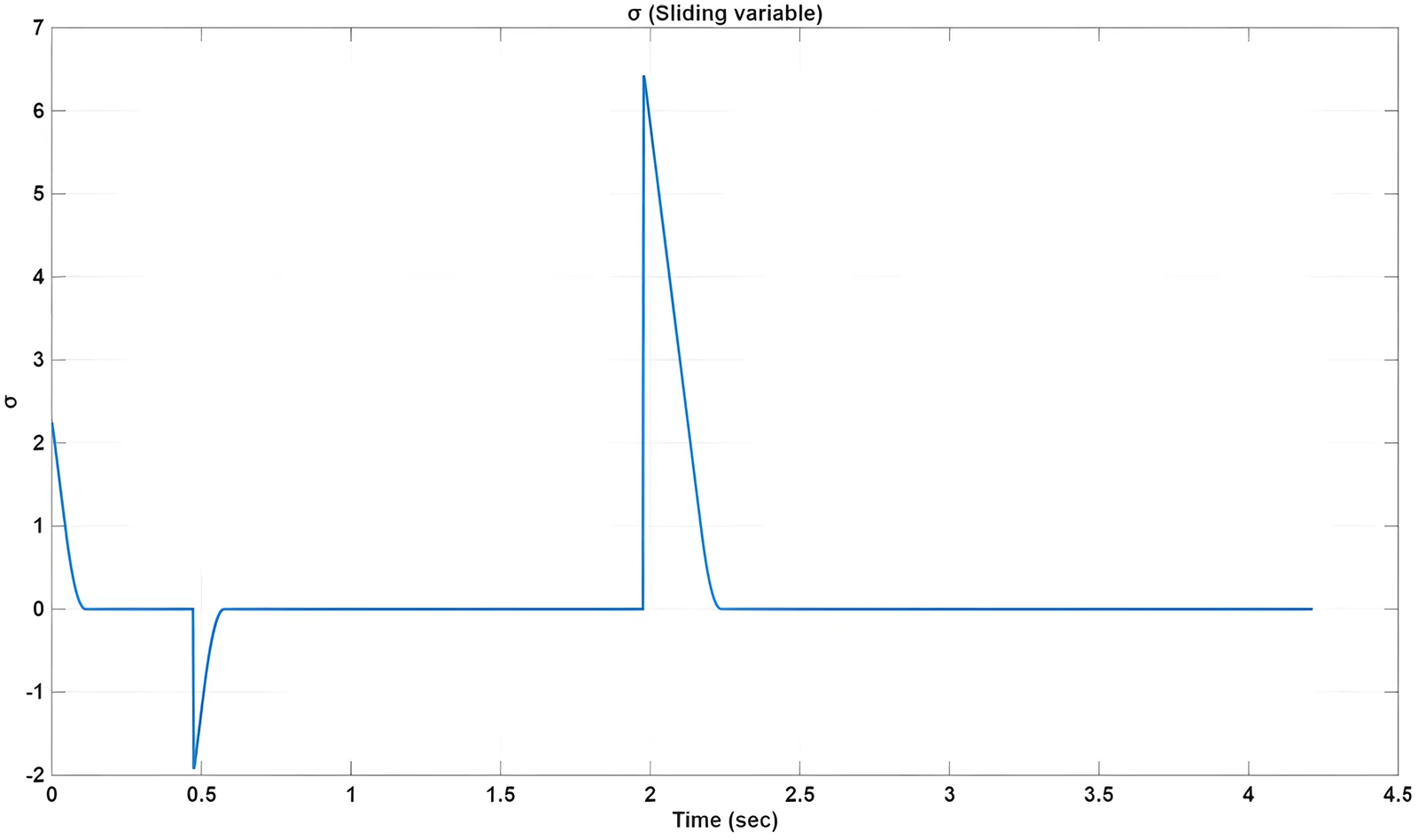
The sliding variable σ on the path with an angle of 3π/4rad.


$$\begin{array}{c} The\;average\;of\;cross-track\;error=\frac{\sum_{i=\text{0}}^{number\;of\;samples-1}\left|e(i\tau )\right|}{number\;of\;samples} \end{array}$$ (23)

Moreover, the angular velocities produced by the proposed controller were much smoother than those produced by the benchmark controller, as shown in Fig 11, Fig 15, and Fig 19. The angular velocity produced by the proposed controller increased rapidly during turns and then returned to zero throughout the straight segments. Conversely, the angular velocity control signal of the benchmark controller fluctuates continuously. This may limit the applicability of the benchmark controller in real-world applications. Conversely, the proposed controller is applicable to actual robots as shown in the next subsection.

As illustrated in Fig 12, Fig 16, and Fig 20, the proposed controller produces very tight turns by a sharp reduction in the linear velocity when the angular velocity is high. However, the benchmark controller did not significantly reduce the linear velocity, and hence, the turns were not tight. However, because of the sharp reduction in the linear velocity, the proposed controller required a longer time to complete the path than the benchmark controller.

In Fig 13, Fig 17, and Fig 21, the sliding variable σ is depicted. As shown, the sliding variable is always pushed to zero in case of any deviation due to sudden changes in the path direction.

Simulations of three paths were conducted to evaluate the performance of the proposed controller using a robot dynamical model with $\mu_{l}=\text{14}.\text{86}s^{-\text{1}}\text{ and }\mu_{r}=\text{13}.\text{03}s^{-\text{1}}$. Additionally, $\mu_{min}=\text{10}s^{-\text{1}}$ and the other controller parameters remain the same as in previous simulations. The first path is circular, formed by a set of 21 waypoints; the second path is ∞ -shaped, also composed of 21 waypoints; and the third path is a right-angle trajectory defined by a set of three waypoints.

As shown in Fig 22, Fig 23, and Fig 24, the controller successfully guides the robot along the desired paths, achieving a convergent cross-track error. As depicted in Fig 25, Fig 26, and Fig 27, spikes appear in the graph of σ, corresponding to sudden directional changes at the waypoints.

**Fig 22:**
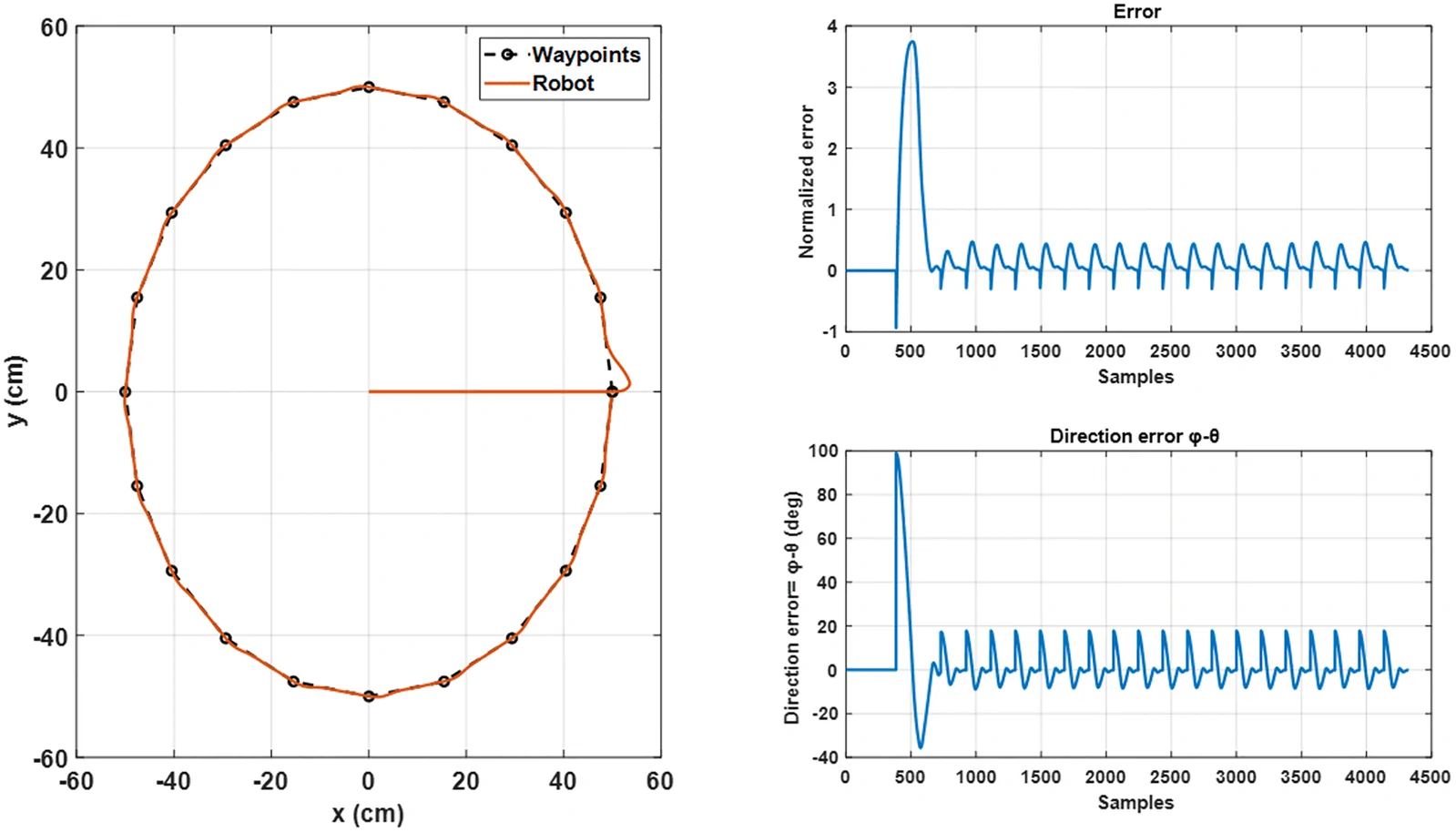
Simulation of a circular path: Error is the normalized error $e_{n}$ and Direction error is φ−θ.

**Fig 23:**
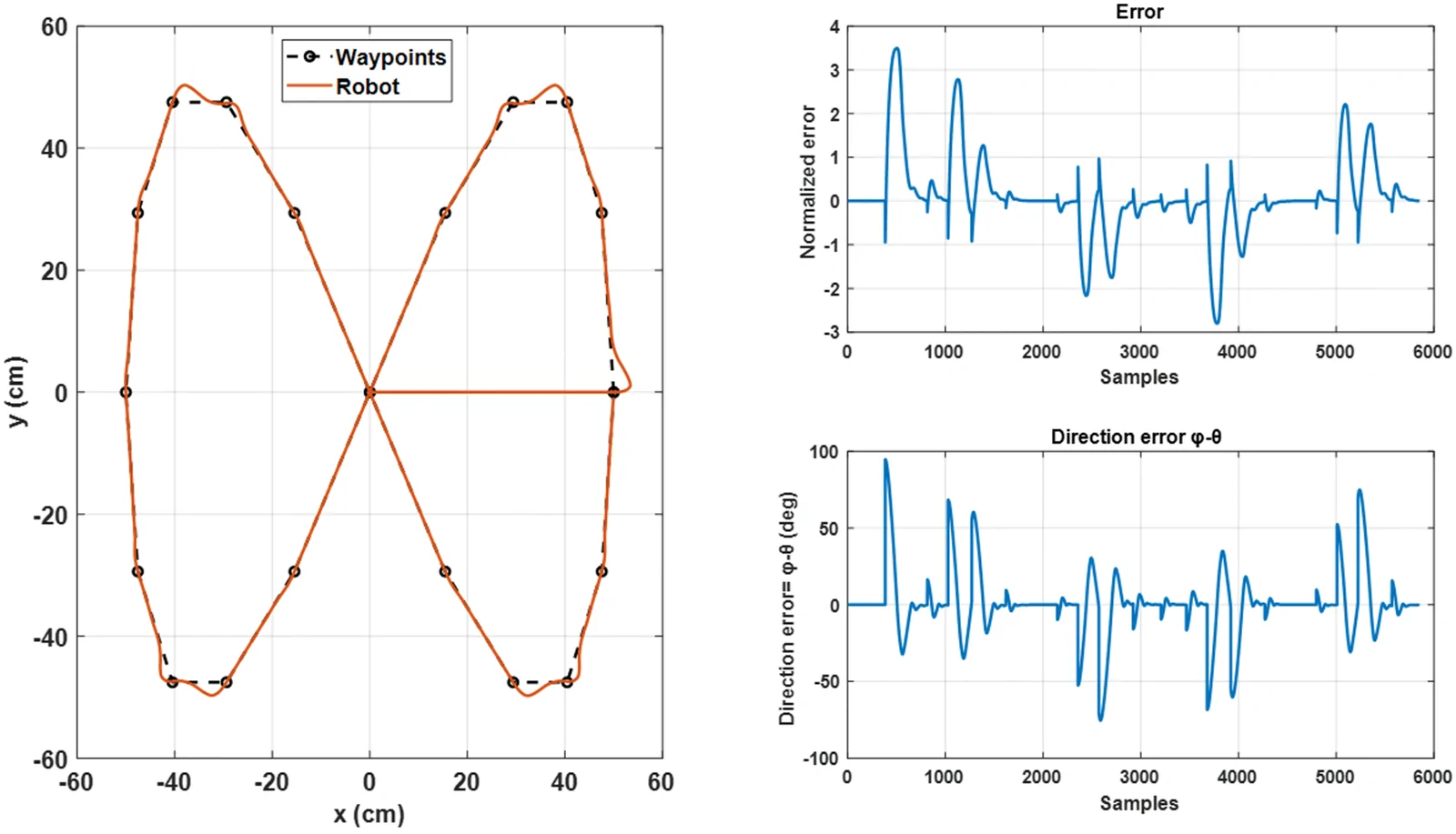
Simulation of an ∞-shaped path: Error is the normalized error $e_{n}$. Direction error is φ−θ.

**Fig 24:**
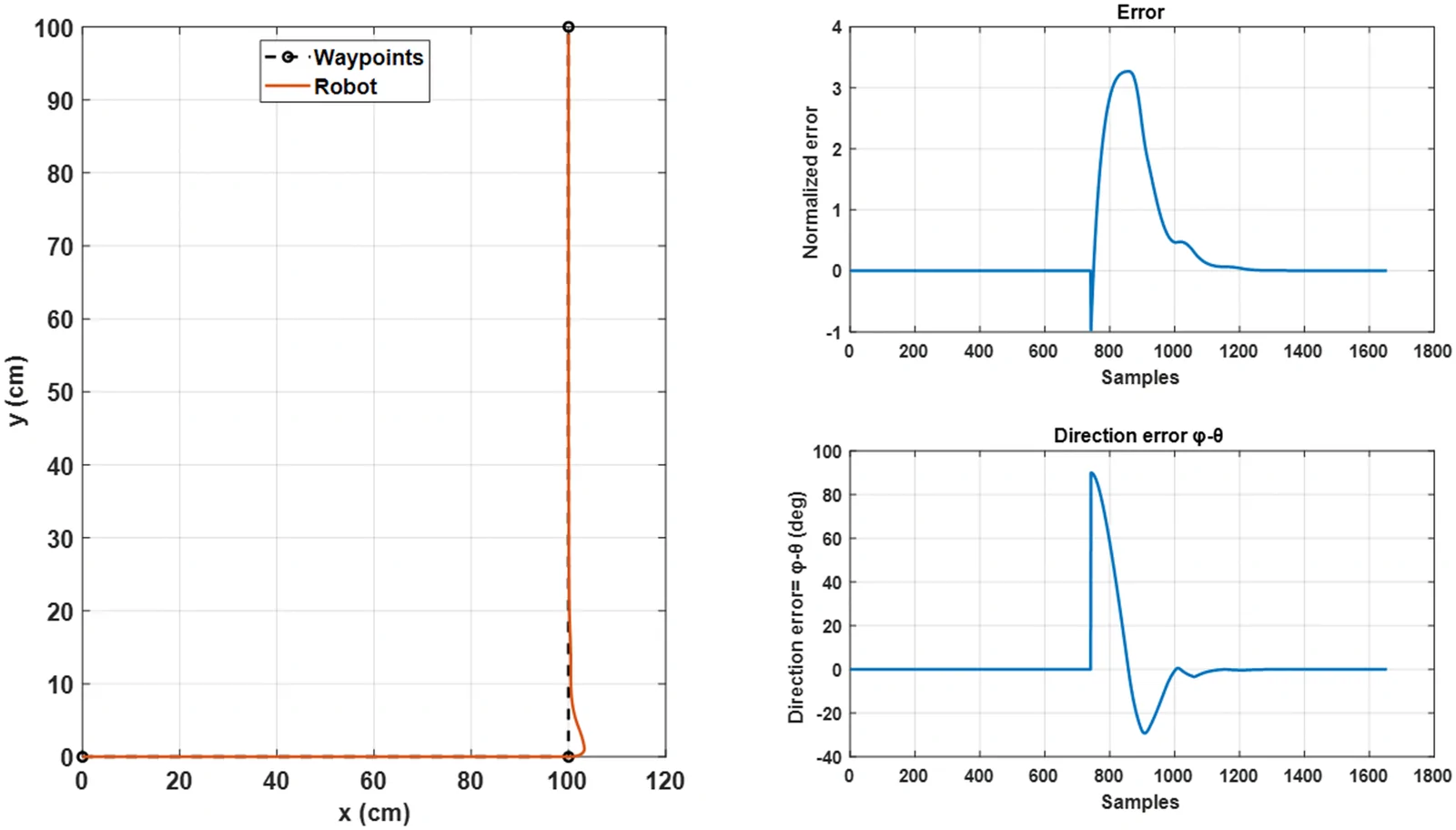
Simulation of a right-angled path.

**Fig 25:**
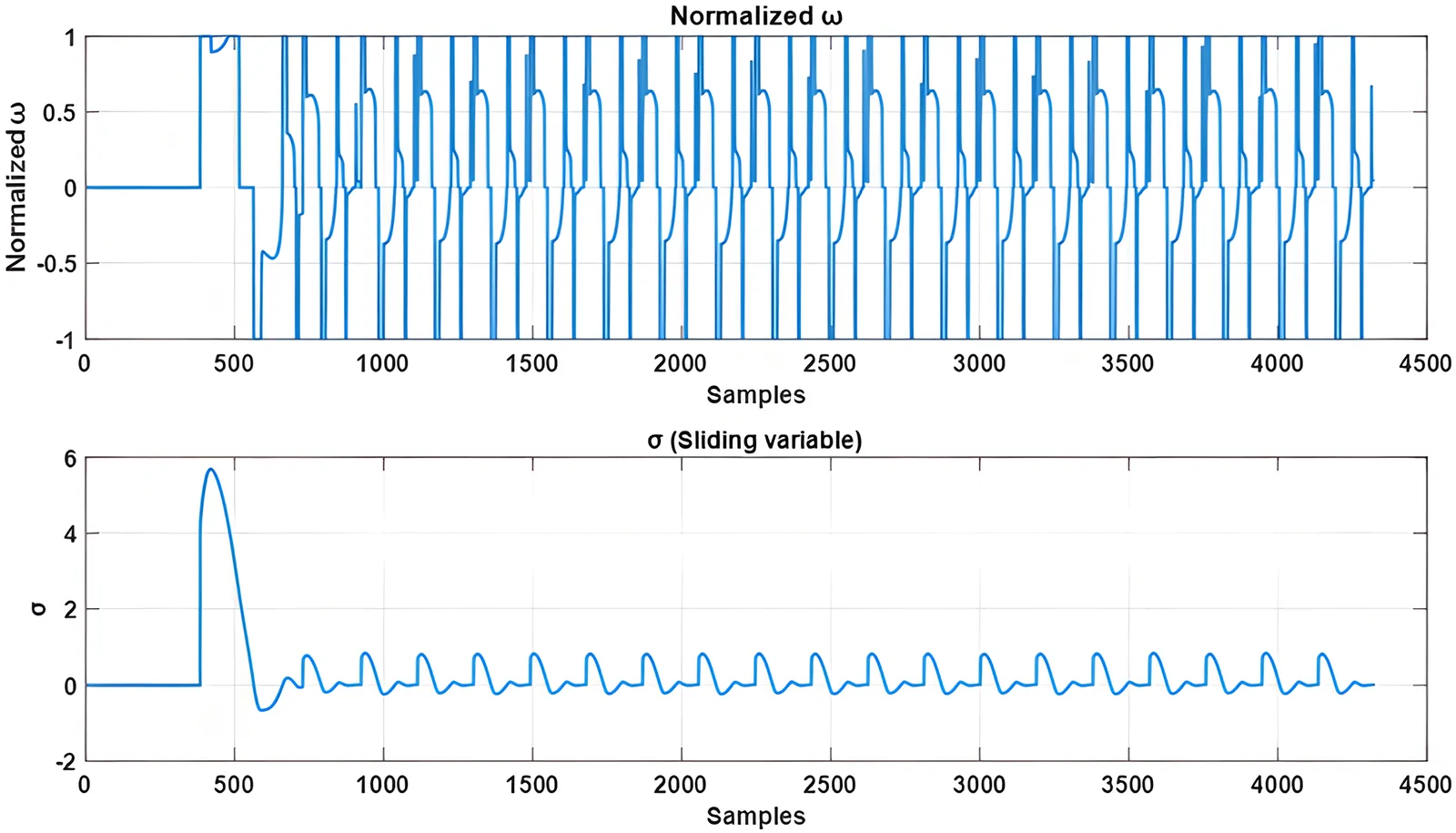
Simulation of a circular path: The control input ($\omega_{n}$) and the sliding variable (σ).

**Fig 26:**
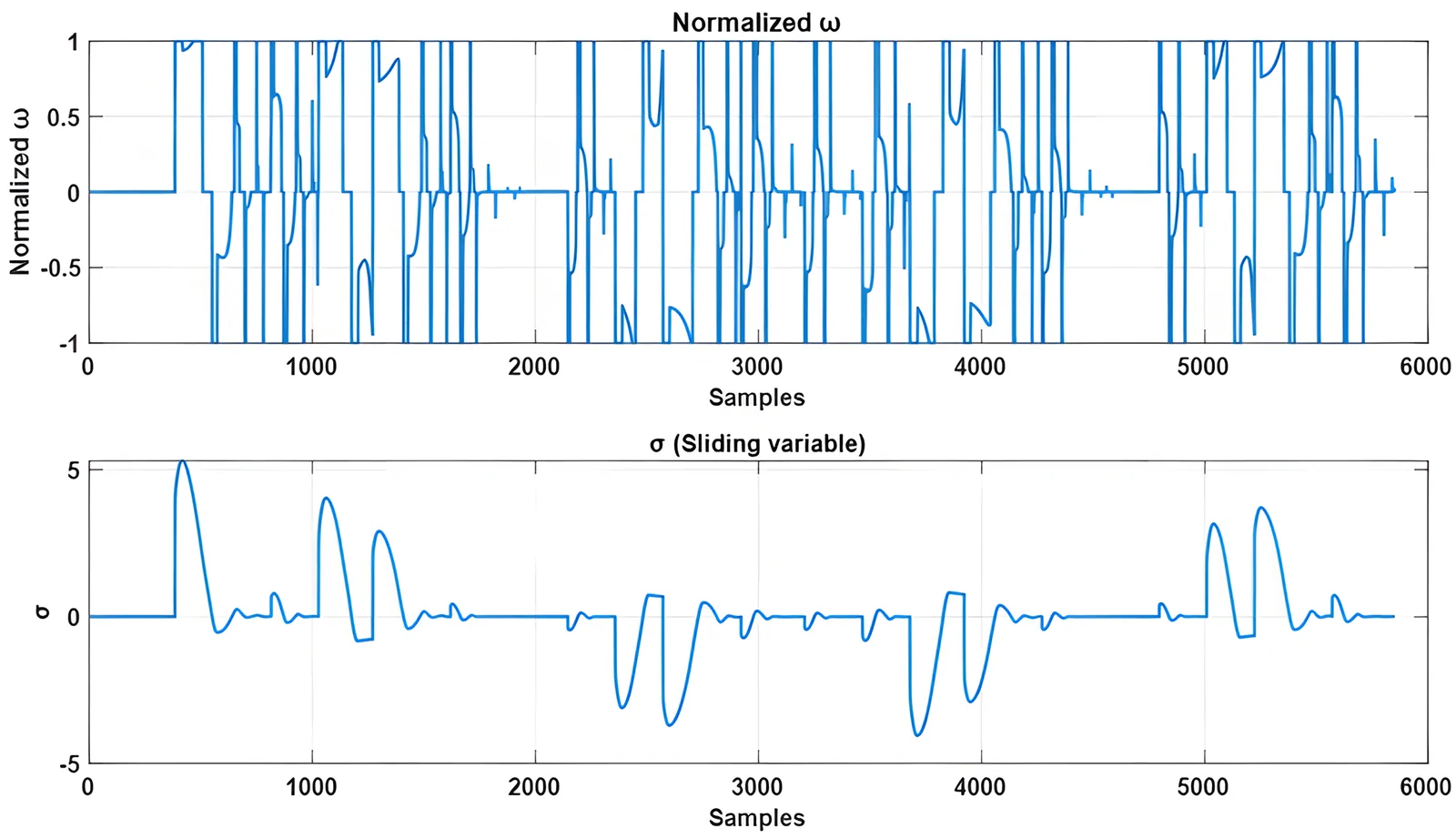
Simulation of an ∞-shaped path: The control input ($\omega_{n}$) and the sliding variable (σ).

**Fig 27:**
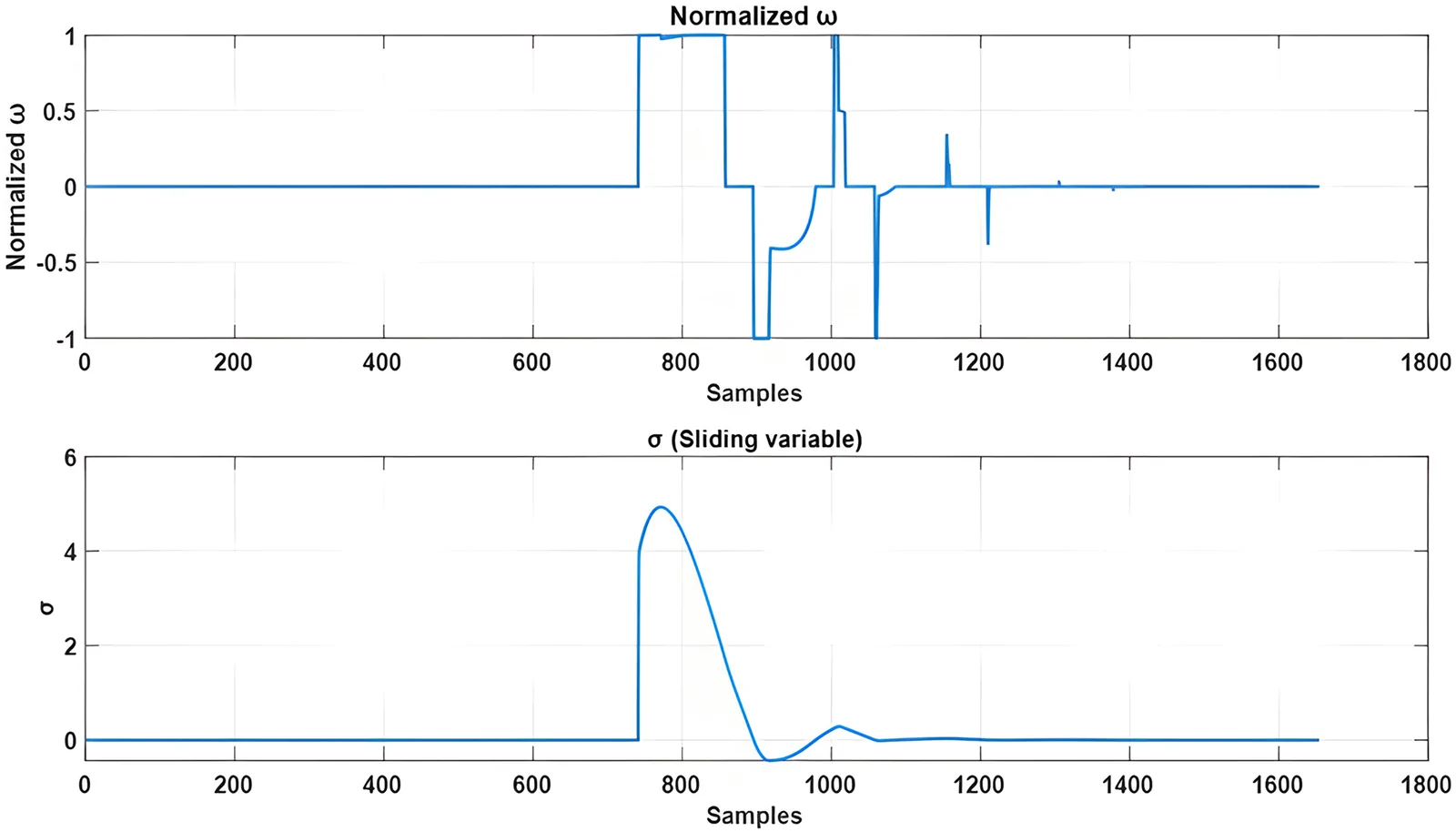
Simulation of a right-angled path: The control input ($\omega_{n}$) and the sliding variable (σ).

### 4.2. Experimental tests

In this section, the proposed control system is tested using a differential drive robot, as shown in Fig 28, to track paths described by sets of waypoints. The robot has two motorized wheels with a quadrature encoder mounted on each wheel. The encoder resolution is 28 pulses/ cm. The encoders are sampled each ${\text{1}\text{0}}^{-\text{4}}$ s. The robot total mass is approximately 0.8 kg. The robot is controlled using an Arduino Due microcontroller. The parameters of the robot are $\omega_{m}=\text{1}\text{0}\text{ rad}/\text{s}$, $\vartheta_{m}=\text{7}\text{0}\text{ cm}/\text{s}$. The robot was initially located at coordinates $\backslash {(}_{p}^{F}x=\text{0}$, $\backslash {(}_{p}^{F}y=\text{0}$, and θ=0 rad. The controller parameters were $e_{m}=\text{1}\text{ cm}$, ${e_{\theta }}_{m}=\pi$/18$\text{ rad},\;\mu_{min}=\text{1}\text{0}\;{\text{s}}^{-\text{1}},\text{ and }\delta =\text{0}.\text{0}\text{1}$. The parameter $d_{tol}$ was set as 1 cm*.* The sampling time of the controller was $\text{2}\times {\text{1}\text{0}}^{-\text{3}}\text{ s}$. It is assumed that the exact parameters of robot dynamics are unknown. Only the lower bound $\mu_{min}$ was estimated. Consequently, the control law in Eq. (21) is used. During the operation of the robot, the data were stored in an array in microcontroller memory. At the end of each path, the collected data was sent to a PC via a Wi-Fi link. During the physical experiments, the current pose of the robot is continuously updated from the encoder data using the discrete-time odometry equations previously established in Eq. (22) in subsection 3.4.

**Fig 28:**
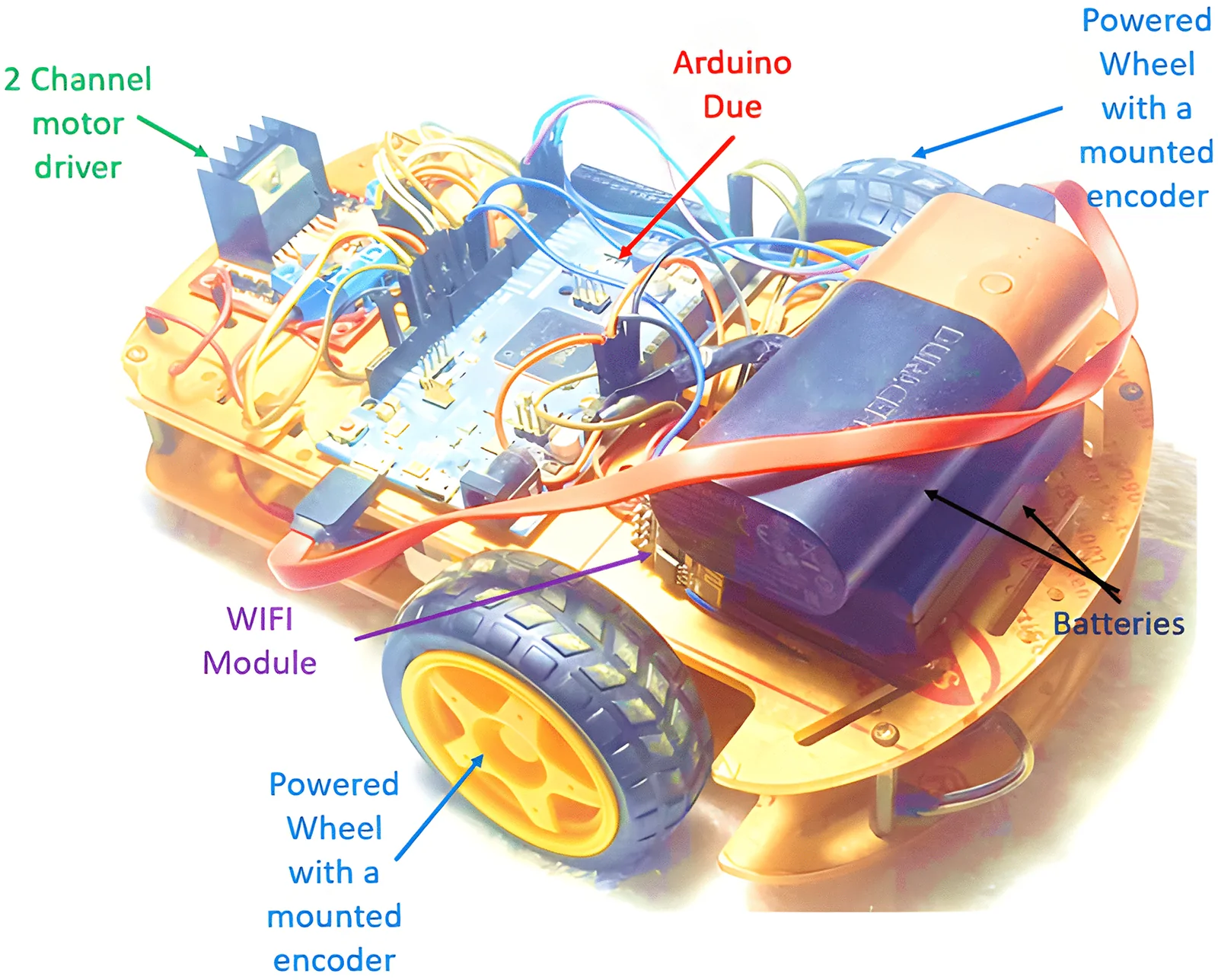
The differential drive robot that was used for the experimental tests.

For a slow-moving robot, slipping can be ignored. Consequently, over short distances, encoders may accumulate only small errors, making the calculated pose nearly identical to the robot’s actual pose. However, in applications involving long distances or higher velocities, additional sensors-such as GPS, lidars, and cameras along with more advanced sensor fusion algorithms, may be required for accurate pose estimation.

In the 1^st^ Experiment, the robot tracked a circular path defined by a set of 21 waypoints. The desired path, actual robot path, normalized error $e_{n}$, and direction error φ−θ in degrees are illustrated in Fig 29. As shown, the initial position of the robot was not at any waypoint. The robot successfully moved to the 1^st^ waypoint and tightly tracked the path. From the results, the controller was able to drive the robot to follow the desired path with a negligible error.

**Fig 29:**
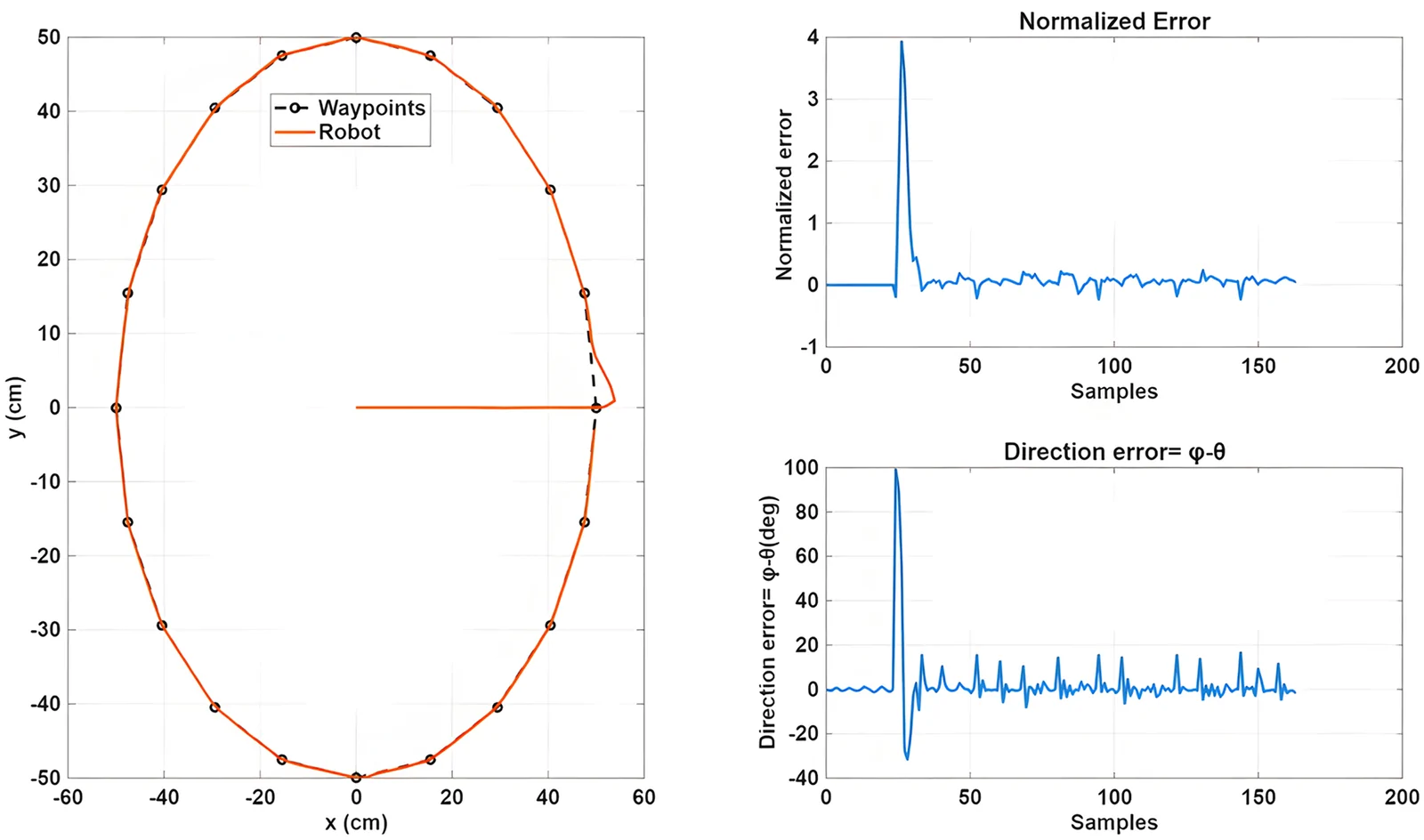
Experiment 1- a circular path: Error is the normalized error $e_{n}$. Direction error is φ−θ**.**

In the 2^nd^ experiment, the robot tracked a ∞-shaped path, as shown in Fig 30. The ∞-shaped path was defined using 21 waypoints. The robot successfully tracked the path following the straight segments connecting the waypoints.

**Fig 30:**
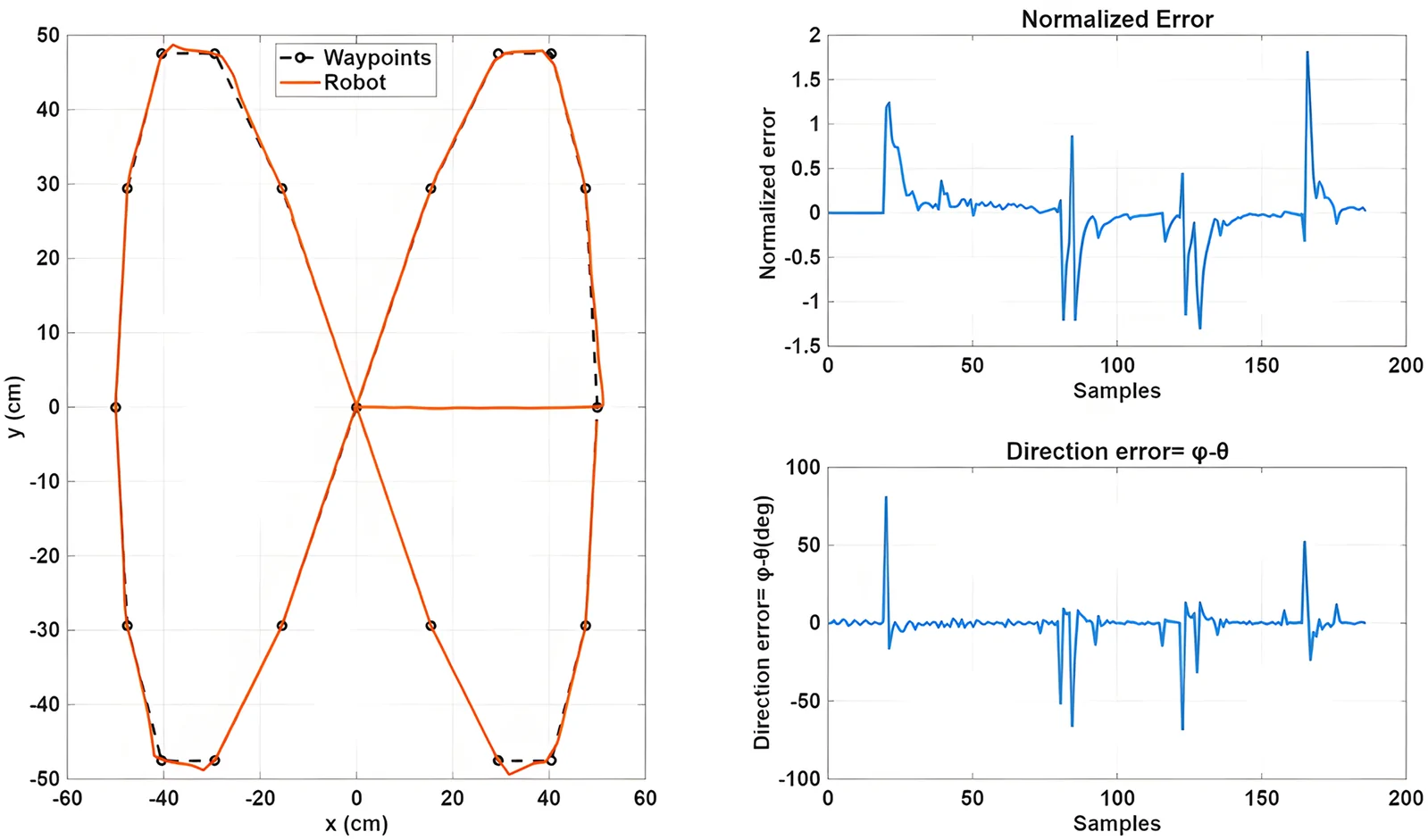
Experiment 2- an ∞-shaped path: Error is the normalized error $e_{n}$. Direction error is φ−θ.

In the 3^rd^ experiment, the robot tracked a path defined by three waypoints with a directional discontinuity at an angle of π/2rad, as shown in Fig 31. The robot successfully performed a tight maneuver to change direction by π/2 rad.

**Fig 31:**
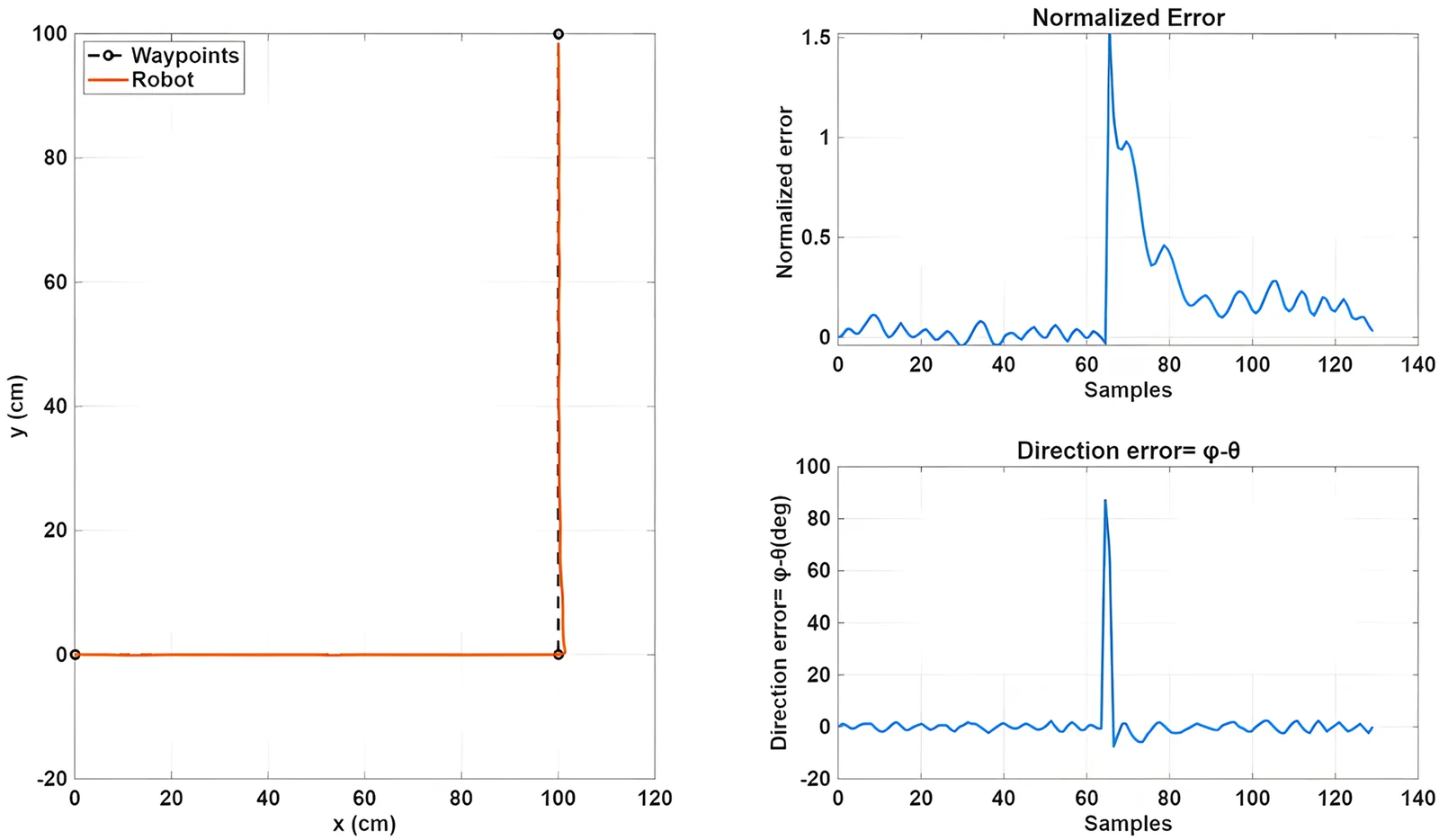
Experiment 3- a right-angled path: Error is the normalized error $e_{n}$. Direction error is φ−θ.

As shown in Fig 32, Fig 33, and Fig 34, the sliding variable σ is pushed to zero except at some points it becomes high because of sudden changes in the direction of paths. However, this variable has some ripples around zero because of the ripples in error due to the ripples in the readings of the encoders and consequently the controller takes corrective actions revealed in the angular velocity graphs.

**Fig 32:**
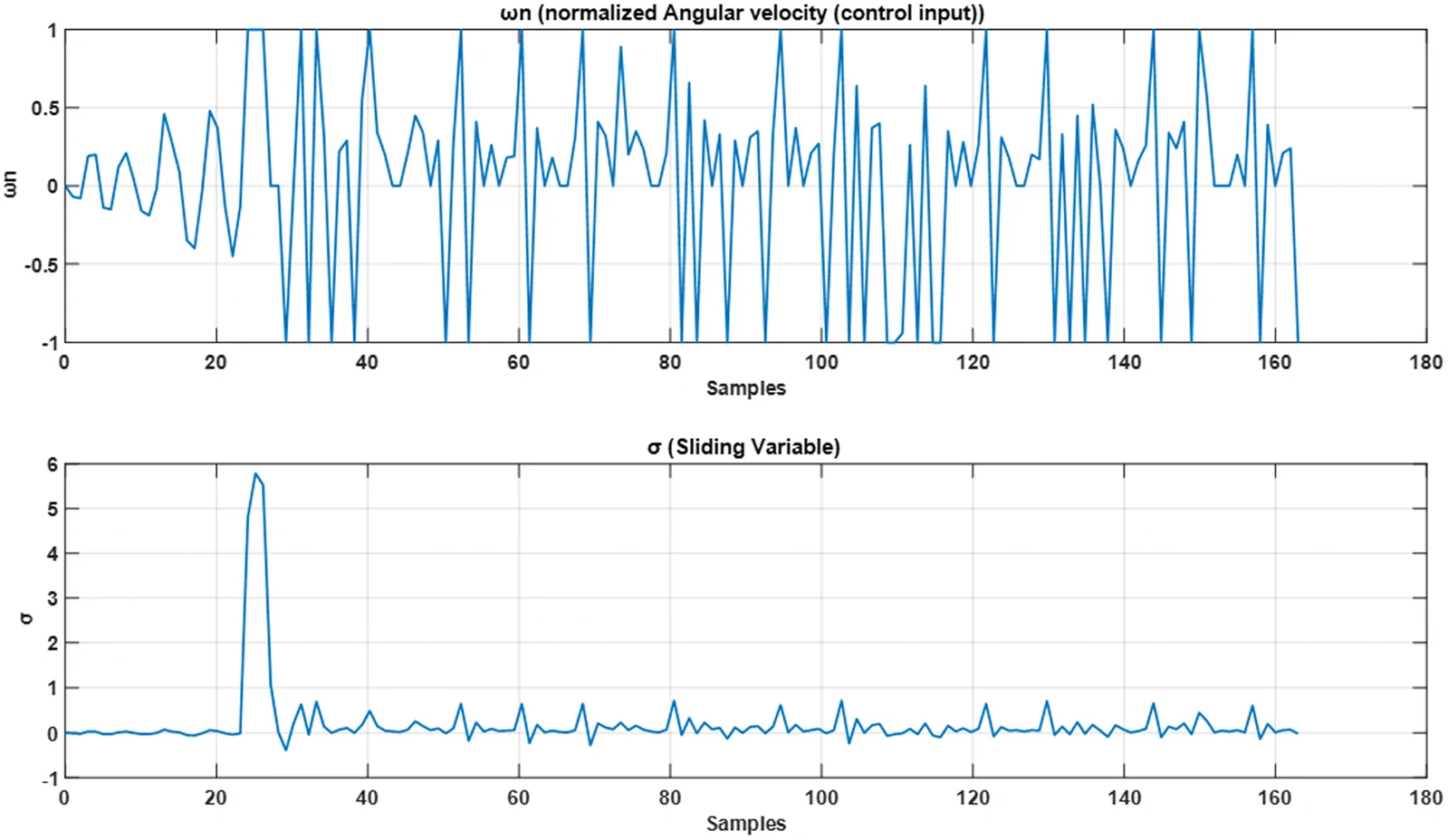
Experiment 1- a circular path: The control input ($\omega_{n}$) and the sliding variable (σ).

**Fig 33:**
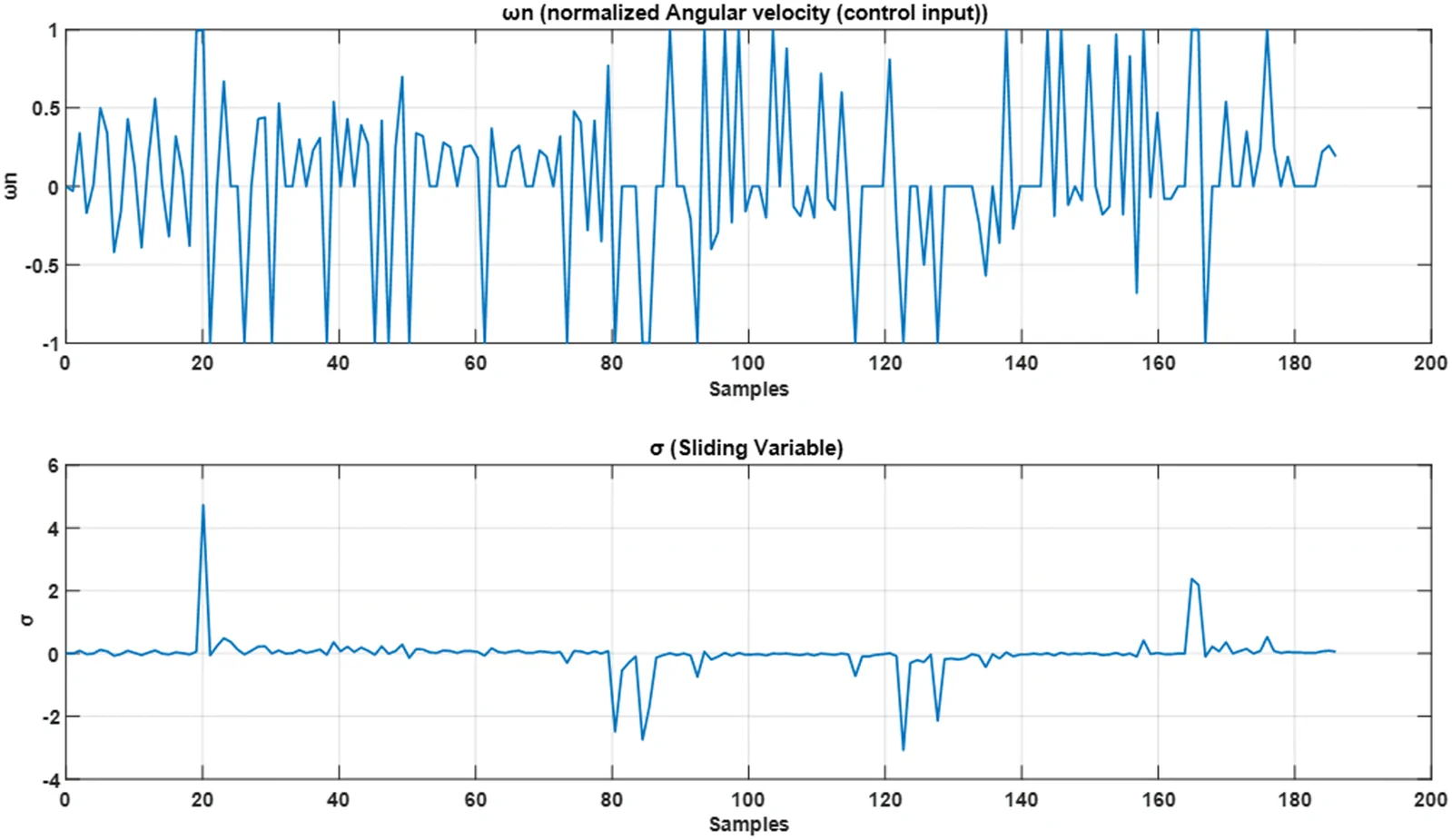
Experiment 2- an ∞-shaped path: The control input ($\omega_{n}$) and the sliding variable (σ).

**Fig 34:**
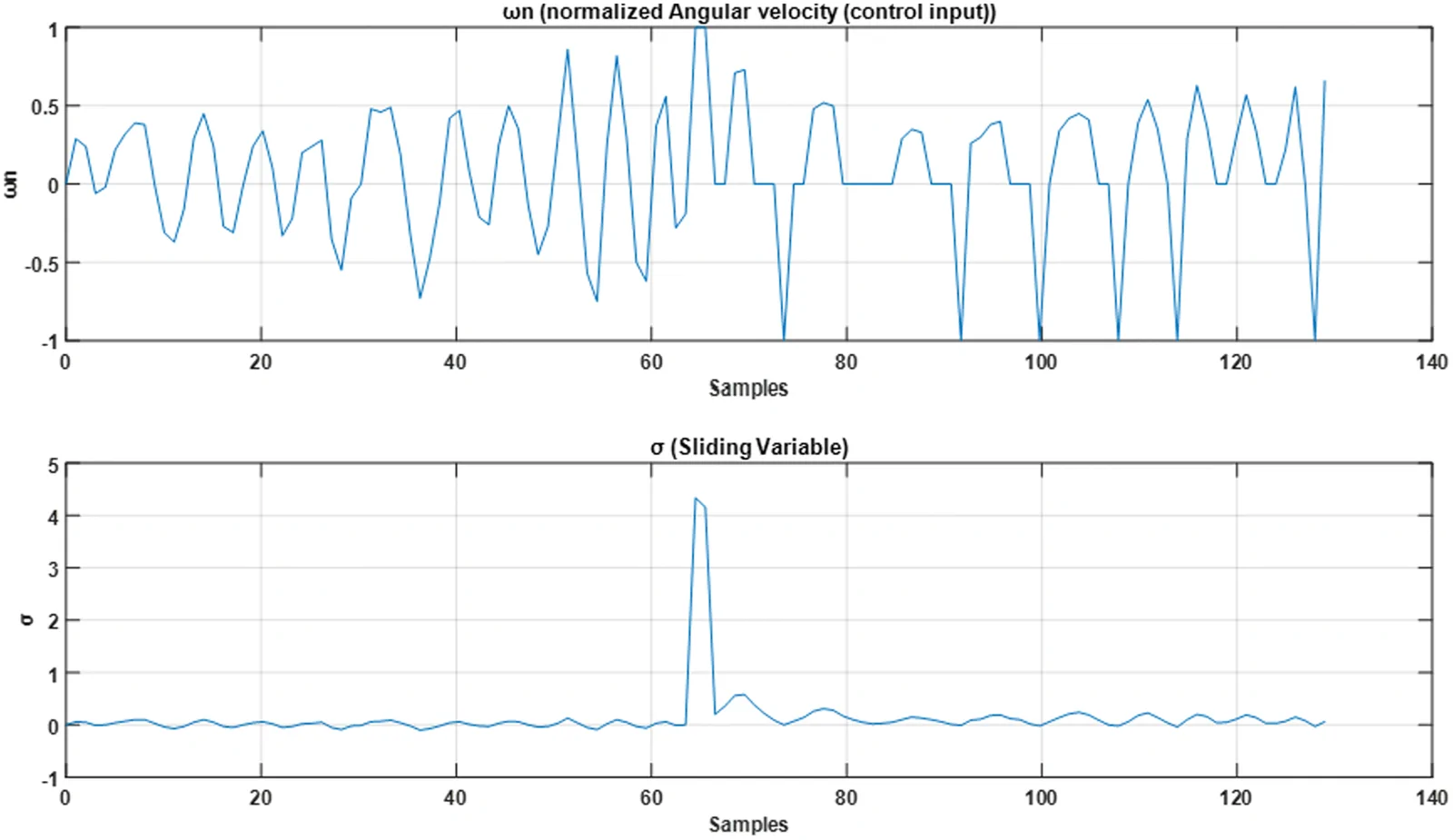
Experiment 3- a right angled path:The control input ($\omega_{n}$) and the sliding variable (σ).

## 5. Conclusion and future work

The main objective of this research is to build a stable controller for a path-tracking system with an arbitrary set of waypoints. A new approach was introduced for a path-tracking control system for differential drive robots. This approach is based on a nonlinear model of the tracking error, namely the cross-track error and angular error shown in Eq. (5) and Eq. (6), respectively. A kinematic model of the cross-track and angular errors was developed in this research, as shown in Eq. (10). Based on the developed error model, a stable SMC was proposed that pushes the error states to the sliding surface σ=0. The stability of this controller is proven by showing that the error approaches zero when the system moves on the sliding surface, that is, σ=0. A control law is introduced to guarantee the reachability of the sliding surface in finite time. The proposed SMC is expressed by Eq. (14). The effect of robot dynamics is investigated, where the Coriolis and centrifugal forces are assumed to be insignificant, as in the case of low velocities, and the center of rotation is sufficiently close to the center of mass. The modified control law in Eq. (21) was introduced to compensate for the dynamics of the robot. This control algorithm was successfully tested through numerical simulations using different sets of waypoints with directional discontinuities and/or sharp turns at angles π/4, π/2, and 3π/4. The effects of the controller parameters were demonstrated by conducting simulations using different values of the parameters. The performance of the proposed controller was compared with that of the controller introduced in [15], which was a modification of the widely used controller introduced in [2]. Simulations show that the proposed controller outperforms the existing control technique for an arbitrary set of waypoints at directional discontinuity and/or sharp turns. In addition, the proposed controller converges tightly to the straight segments between waypoints, achieving smaller values of average and maximum errors than those of the existing control technique. Subsequently, the proposed controller was successfully verified by using a differential drive robot with different waypoint sets. Experiments using a real robot demonstrate the applicability and tracking accuracy of the proposed controller.

**Future Work:**

Based on this research, several open research questions remain to be addressed in the future. Some of these points are as follows:

- Finding a suitable nonlinear sliding surface for finite-time error convergence for the error modeled by Eq. (10).
- Studying the performance improvements that can be achieved by using second-order sliding mode control.
- Trajectory-tracking applications require a desired time profile: in this case, it may be necessary to derive a modified error model to address timing requirements.
- Autonomous car-like vehicles with front-wheel steering system: the model introduced in Eq. (10) is used for the differential drive steering system. A new model may be required to address the car-like steering systems. Consequently, a suitable sliding surface is required to ensure vanishing errors.
- Autonomous aerial drones: based on the kinematics of drones, a new mathematical model for posing errors is required. Based on this model, a suitable sliding surface must be chosen to eliminate posing errors over time. Therefore, a control law is required to consider this new sliding surface and drone dynamics.
- Vehicle dynamics under Coriolis and centrifugal forces: in this paper, the Coriolis and centrifugal forces are ignored, assuming low velocities and that the center of rotation is sufficiently close to the center of mass. These assumptions are valid for several robotic applications in workshops, hospitals, and warehouses. However, high speeds are required for autonomous street vehicles, and fast robots are even preferable in some large warehouses. In such cases, Coriolis and centrifugal forces must be considered. Consequently, the introduced control law must be modified to ensure the reachability of suitable sliding surfaces.
- Diverse environmental scenarios and sensor noise: in real-world robotic applications, path-tracking is often subjected to uneven terrains, variable surface friction, and noisy sensor measurements. Future studies will focus on evaluating the robustness of the proposed control algorithm under more diverse environmental scenarios and varying levels of encoder noise, potentially incorporating advanced noise filtering or sensor fusion techniques to further validate its practical reliability.
